# Visual Function is Gradually Restored During Retina Regeneration in Adult Zebrafish

**DOI:** 10.3389/fcell.2021.831322

**Published:** 2022-02-01

**Authors:** Juliane Hammer, Paul Röppenack, Sarah Yousuf, Christian Schnabel, Anke Weber, Daniela Zöller, Edmund Koch, Stefan Hans, Michael Brand

**Affiliations:** ^1^ CRTD—Center for Regenerative Therapies at TU Dresden, Dresden, Germany; ^2^ Clinical Sensoring and Monitoring, Department of Anesthesiology and Intensive Care Medicine, Carl Gustav Carus Faculty of Medicine, TU Dresden, Dresden, Germany

**Keywords:** zebrafish, retina, regeneration, functional recovery, visually-mediated behavior, optokinetic response, social preference test, optical coherence tomography

## Abstract

In comparison to mammals, zebrafish are able to regenerate many organs and tissues, including the central nervous system (CNS). Within the CNS-derived neural retina, light lesions result in a loss of photoreceptors and the subsequent activation of Müller glia, the retinal stem cells. Müller glia-derived progenitors differentiate and eventually restore the anatomical tissue architecture within 4 weeks. However, little is known about how light lesions impair vision functionally, as well as how and to what extent visual function is restored during the course of regeneration, in particular in adult animals. Here, we applied quantitative behavioral assays to assess restoration of visual function during homeostasis and regeneration in adult zebrafish. We developed a novel vision-dependent social preference test, and show that vision is massively impaired early after lesion, but is restored to pre-lesion levels within 7 days after lesion. Furthermore, we employed a quantitative optokinetic response assay with different degrees of difficulty, similar to vision tests in humans. We found that vision for easy conditions with high contrast and low level of detail, as well as color vision, was restored around 7–10 days post lesion. Vision under more demanding conditions, with low contrast and high level of detail, was regained only later from 14 days post lesion onwards. Taken together, we conclude that vision based on contrast sensitivity, spatial resolution and the perception of colors is restored after light lesion in adult zebrafish in a gradual manner.

## 1 Introduction

Vision is one of the most important senses in all vertebrate species, particularly in humans. According to the World Health Organization, approx. 2.2 billion people are currently affected by visual impairment or even blindness (World Health Organization, 2019). Available treatments and therapies are very limited allowing in most cases only a deferral of the underlying etiopathology. Various attempts have been made to prevent or restore vision loss using prosthetic devices, gene therapy, optogenetics as well as cell transplantations (Salman et al., 2021). Another promising approach aims to rekindle the innate property of the retina to self-repair which has been observed in several vertebrate species. Mammals, including humans, display only limited regenerative capacities upon retinal injury. Müller glia, the retinal stem cells, remain either quiescent or respond to injury with reactive gliosis (Karl and Reh, 2010). Several successful attempts have been recently made enabling mammalian Müller glia to evade quiescence and stimulating a regenerative response to ameliorate the etiopathology (reviewed in (Salman et al., 2021)). However, Müller glia-based regeneration is still insufficient in mammals. In stark contrast, fish are highly regenerative organisms that are able to regenerate the retina, as well as other tissues and organs, upon injury without any further stimulation (Wan and Goldman, 2016). Following retinal insult, damaged neurons undergo cell death and activate Müller glia which reenter the cell cycle resulting in the production of multipotent progenitors. Following further subsequent cell divisions, the progenitor cells differentiate into all retinal cell types replacing the lost cells (Goldman, 2014). To address regeneration in the adult fish retina, various lesion models have been established, including intense light, needle poke or surgery, intravitreal injections of or soaking in neurotoxins as well as Nitroreductase-induced targeted cell ablation (Hitchcock et al., 1992; Senut et al., 2004; Vihtelic et al., 2006; Fimbel et al., 2007; Mensinger and Powers, 2007; Sherpa et al., 2008; Montgomery et al., 2010; Fraser et al., 2013; Weber et al., 2013; Maurer et al., 2014; Powell et al., 2016; Wang et al., 2017). Although full regeneration requires both structure and function, to date only a few studies assess functional recovery after retinal lesion (Angueyra and Kindt, 2018). For instance, it was shown in goldfish that the dorsal light reflex is recovered within 30 weeks after administration of the neurotoxin Ouabain whereas the recovery takes approx. 80 days after surgical lesion (Mensinger and Powers, 1999, Mensinger and Powers, 2007). Using a vision-dependent place-preference test, Sherpa and colleagues applied an escape response test and reported restoration of functional vision at 60 days after a mild neurotoxic lesion and at 100 days after whole retina destruction (Sherpa et al., 2008, Sherpa et al., 2014). Finally, in a N-methyl-N-nitrosourea-induced retinal degeneration model it was shown that the optokinetic response, a reflexive and stereotypic eye movement, is restored within 15 days after lesion (Maurer et al., 2014). Taken together, these studies indicate that the kinetic of vision recovery can be addressed functionally in the course of regeneration and is strongly dependent on the applied lesion model. However, the systematic application of behavioral assays to assess the degree of functional recovery is currently still largely missing in zebrafish.

Here, we combined the analysis of functional and morphological regeneration in a light lesion paradigm that specifically ablates photoreceptors in adult zebrafish. To this end, various vision-dependent behavioral tests were performed before lesion and during the course of regeneration. Using a novel vision-dependent social preference test, we find that basic visual properties are already restored within 7 days after lesion. In addition, using a two level optokinetic response (OKR)-based vision test, we demonstrate that color vision as well as vision under easy conditions, with high contrast and low level of detail, is restored within 10 days. Vision under more demanding conditions, with low contrast and high level of detail, is restored in the subsequent days. Comparison of the obtained behavioral data with morphological data using classical HE stainings as well as improved *in vivo* optical coherence tomography (OCT) imaging reveals a close correlation between functional and morphological recovery, indicating that functional recovery is already observed before the completion of morphological restoration. Together, our data provide evidence for gradual recovery of visual performance within 28 days after light lesion in adult zebrafish. We foresee that our applied methodology will allow assessing visual function in zebrafish in the context of other lesion paradigms or disease models in great detail and moreover enable their subsequent direct comparison.

## 2 Materials and Methods

### 2.1 Fish Maintenance

Fish were kept under standard conditions and maintained at 26°C on a 14 h light, 10 h dark cycle as previously described (Brand et al., 2002). All experimental fish were from the wildtype AB genetic background and between 6 and 10 months of age. Both female and male fish were used in equal ratios in all experiments. No gender difference was observed in any of the experiments. To keep track of individual fish during the course of the experiments, pairs of fish (one female, one male) were kept together in separate cages. Behavioral experiments were performed during the light cycle at the same time of the day for each individual test to eliminate any interference of the circadian clock.

### 2.2 Light Lesion

Light lesions were performed as described previously ((Weber et al., 2013); diffuse light lesion). In brief, fish were dark-adapted for 48 h prior to the lesion to ensure a maximum effect on the photoreceptor cells. During the lesion, a pair of freely swimming fish was exposed to very bright light (approx. 200.000 lux, EXFO X-Cite 120 W metal halide lamp) for 30 min. The exposure of fish to very bright light results in a specific and highly reproducible ablation of photoreceptors. More specifically, all rods and cones are ablated in the central part of the retina whereas mostly UV and blue cones are lost in more peripheral regions leaving all other photoreceptor cell types intact. In the uppermost dorsal and ventral regions, all photoreceptors are largely unaffected by the lesion (Weber et al., 2013). Subsequently, all fish were returned to the system for recovery under normal light conditions.

### 2.3 Dark-Light Preference Test

**FIGURE 1:**
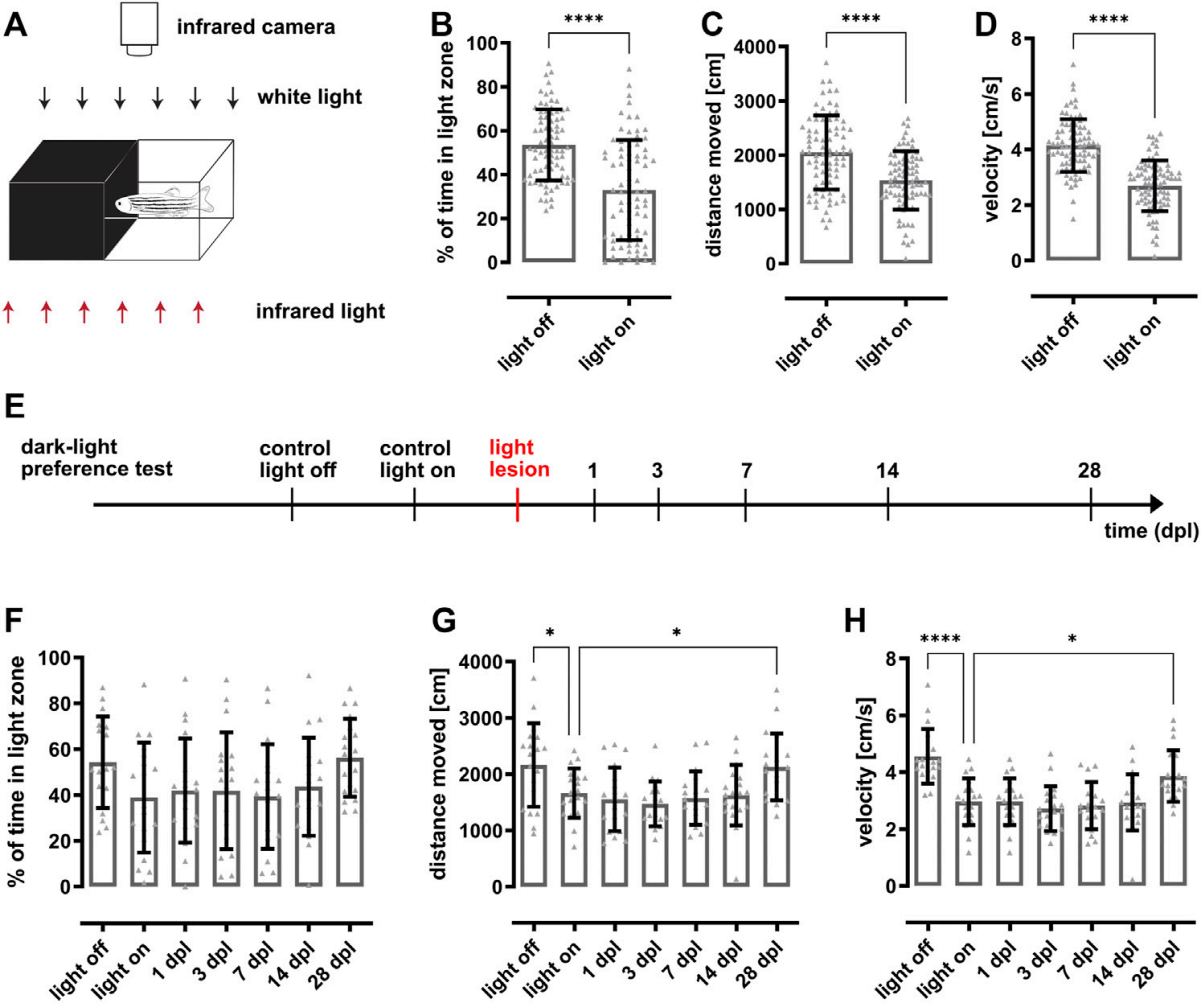
Dark-light preference behavior is statistically significant but highly variable impeding assessment of vision loss and recovery after light lesion. **(A)** Scheme of the dark-light preference setup. The individual compartments of the custom-made arena consist of infrared-transparent material. One half is made from clear, the other half made from black plastic establishing a light and dark zone, respectively. The test fish can freely swim within the whole compartment, which can be illuminated with white light from the top (85 lux). In addition, the arena is illuminated from an infrared light source located beneath the arena and recorded from the top using an infrared-sensitive camera. **(B)** Quantification reveals that the time fish spent in the light zone is significantly higher in the light off condition (53.6 ± 16.1%) compared to the light on condition (32.9 ± 22.7%). Quantifications of the distance moved **(C)** and the corresponding velocity **(D)** show that the fish swam significantly less (light off: 2060 ± 683 cm; light on: 1537 ± 543 cm) and slower (light off: 4.2 ± 0.9 cm/s; light on: 2.7 ± 0.9 cm/s) in illuminated condition. **(E)** Experimental timeline. Dark-light preference recordings were performed in light on and light off conditions prior to light lesion as well as in light on conditions at 1, 3, 7, 14, and 28 days post lesion (dpl). **(F)** Quantification of the time spent in the light zone reveals that fish do not change their preference for the dark or light zone after light lesion (light off: 54.3 ± 19.5%, light on: 38.9 ± 23.4%, 1 dpl: 41.9 ± 22.1%, 3 dpl: 41.8 ± 24.9%, 7 dpl: 39.3 ± 22.2%, 14 dpl: 43.6 ± 20.9%, 28 dpl: 56.3 ± 16.6%). Similarly, quantifications of the distance moved **(G)** and velocity **(H)** show no lesion-dependent alterations. Statistics: all data are represented as mean ± SD, unpaired t-test **(B–D)**, one-way ANOVA with Dunnett’s multiple comparison test against control **(F–H)**, *p* < 0.05 (*); 0.01 (**); 0.001 (***) or 0.0001 (****).

A custom-made arena (Noldus Information Technology) was used to assess the dark-light preference behavior of adult zebrafish (Figure 1A). The arena is divided into ten individual compartments (10 × 20 cm) with one half made from black and the other from clear plastic. The whole arena is transparent to infrared light. The walls of the clear part of the arena are covered with white foil preventing that fish interact with their conspecifics. To ensure reproducible light conditions for all experiments, the arena was placed inside the ZebraCube (ViewPoint Behavior Technology). For “light on” conditions, nine internal LED clusters generated a homogenous illumination with white light of 85 lux. In addition, the arena was homogenously illuminated via the inbuilt infrared light source at the bottom of the ZebraCube. Fish were recorded using the acA1300-60 NIR camera (Basler AG) equipped with an 111.8″ F1.6/4.4–11 mm objective (Kowa Optimed Deutschland GmbH) and an ES43 RG 850 infrared filter (Heliopan Lichtfilter-Technik Summer GmbH and Co KG) placed above the arena. For all experiments, each compartment of the arena was filled with fresh system water and occupied with one test animal. Subsequently, individual fish in the ten compartments were recorded for 10 min simultaneously after an acquaintance period of 5 min. Recordings in the dark were performed prior to the actual examination of the dark-light preference behavior to exclude an inherent preference of the animal to either part of the compartment independent of the illumination. All recordings were performed between 9 and 12 am with feedings taking place 1 h prior to the experiment. Recordings and subsequent analyses were performed using the EthoVision^®^ XT software (Noldus Information Technology, version 11.5). Parameters of interest comprised: time spent in light zone (in %), distance moved (in cm) and velocity (in cm/s). In addition, heat maps representing the average location of the fish were generated. The experiment was performed with 79 fish for the establishment of the paradigm (Figures 1B–D) and 19 fish for the regeneration time course (Figures 1F–H).

### 2.4 Social Preference Test

**FIGURE 2:**
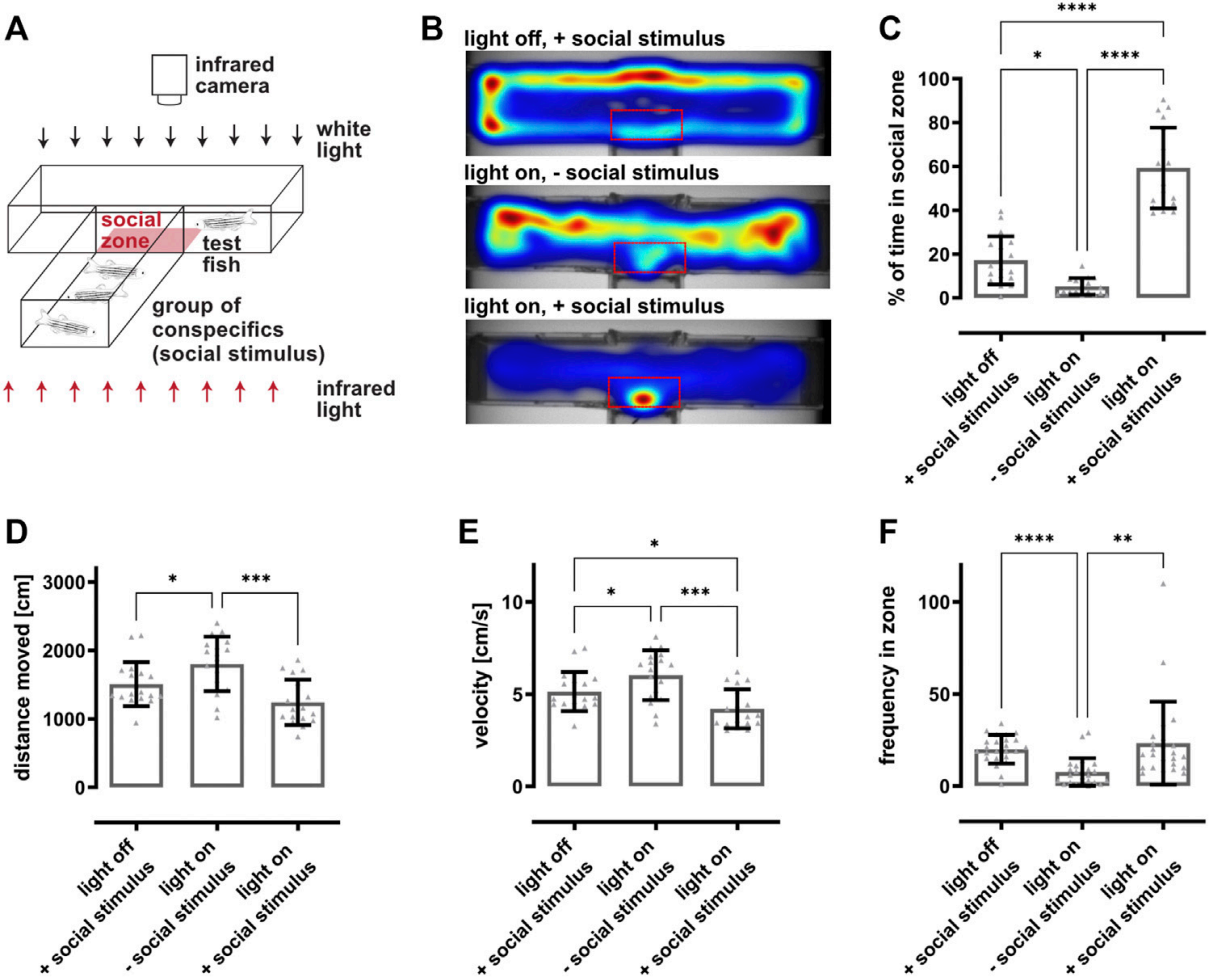
Social preference behavior is dependent on vision. **(A)** Scheme of the social preference setup. A custom-made T-shaped arena is divided into two completely separated compartments. The lower arm harbors a group of eight fish representing the social stimulus, whereas a single test fish is placed into the upper, perpendicular arm. A transparent window is the only non-opaque interface between the two compartments. The movement of the test fish within the compartment is recorded from the top using an infrared-sensitive camera and an infrared light source placed beneath. The zone in front of the window is defined as social zone (red box) and used for quantifications. **(B)** Representative heat maps indicate the test fish’s position in the compartment as a function of the condition. In dark conditions and in presence of a social stimulus (light off, + social stimulus), fish stayed in close proximity to the borders of the compartment. In illuminated conditions and absence of a social stimulus (light on, − social stimulus), fish were freely roaming within the whole compartment. In contrast, in illuminated conditions and presence of a social stimulus (light on, + social stimulus), fish spent a significant amount of time within the social zone. **(C)** Quantification of the time spent in the social zone revealed that fish spent significantly less time in the social zone in dark conditions (17.1 ± 10.6%) or in illuminated conditions in the absence of a social stimulus (5.3 ± 3.7%) as compared to light on conditions with a social stimulus (59.3 ± 17.9%). **(D)** Quantification of the distance moved showed that fish swam slightly more in illuminated conditions in the absence of a social stimulus (1804 ± 387 cm) in comparison to dark conditions (1507 ± 313 cm) or illuminated conditions with a social stimulus present (1243 ± 321 cm). **(E)** Quantification of the velocity revealed that the test fish swam faster in illuminated conditions in the absence of a social stimulus (6 ± 1.3 cm/s) compared to dark conditions (5.1 ± 1 cm/s) or in illuminated conditions in the presence of a social stimulus (4.2 ± 1 cm/s). **(F)** Quantification of the frequency of the fish entering the social zone showed that the test fish entered the social zone significantly more often in dark conditions (20 ± 8) or in illuminated conditions with a social stimulus present (24 ± 24) compared to illuminated conditions without social stimulus (8 ± 7). Statistics: all data are represented as mean ± SD, one-way ANOVA with Tukey’s multiple comparison test, *p* < 0.05 (*); 0.01 (**); 0.001 (***) or 0.0001 (****).

**FIGURE 3:**
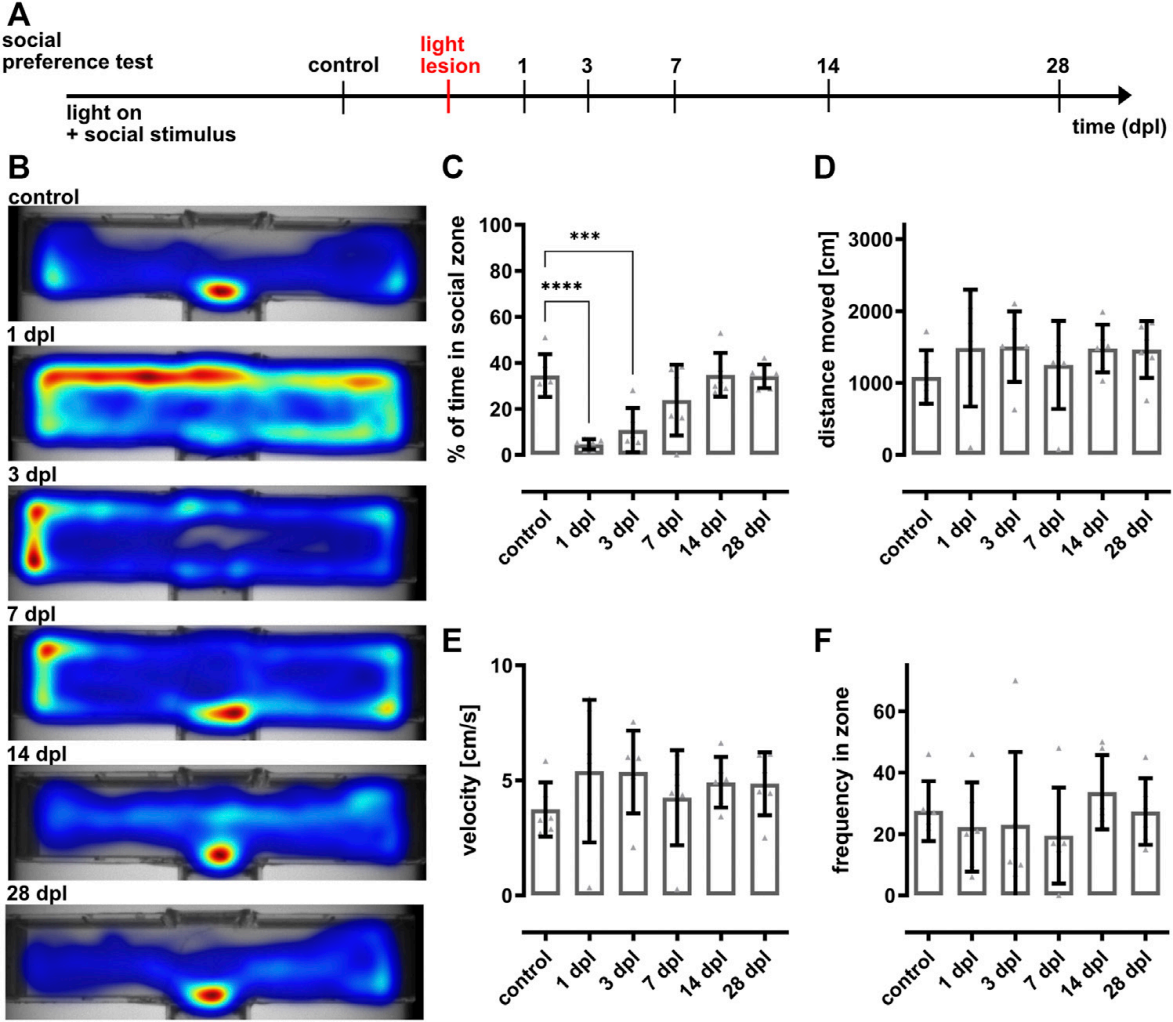
The social preference test reveals recovery of vision within 7 days after light lesion. **(A)** Experimental timeline. Recordings were performed in light on conditions in presence of a social stimulus before light lesion (control) and at 1, 3, 7, 14, and 28 days post lesion (dpl). **(B)** Heat maps of one representative individual fish show that the time spent in the social zone decreases significantly following light lesion and returned to baseline from 7 dpl onwards. **(C)** Quantification of the time spent in the social zone confirms a significant decrease at 1 and 3 dpl (4.6 ± 2% and 10.8 ± 8.8%) and the return to control levels (34.5 ± 8.4%) after 7 dpl (7 dpl: 23.8 ± 14%, 14 dpl: 34.8 ± 8.7%, 28 dpl: 34.1 ± 4.7%). Quantifications of the distance moved **(D)**, velocity **(E)** and frequency in zone **(F)** did not reveal any significant changes within the observed timeframe. Statistics: all data are represented as mean ± SD, one-way ANOVA with Dunnett’s multiple comparison test against control, *p* < 0.05 (*); 0.01 (**); 0.001 (***) or 0.0001 (****).

A customized T-shaped arena was used to analyze vision-dependent recognition of conspecifics making use of the zebrafish inherent preference to socialize (Engeszer et al., 2007; Suriyampola et al., 2016; Orger and De Polavieja, 2017). A group of eight fish with an equal sex ratio was placed into the lower arm of the arena serving as social stimulus while a test fish was put into the separated upper arm (10 × 50 cm) (Figure 2A). The walls of the box are opaque except for a small window at the intersection between the upper and lower arm. A rectangular zone (5 × 9 cm) directly in front of the window was defined as social zone. The same illumination and recording setup described for the dark-light preference test was used (see 3.3). Fish were allowed to get acquainted within the arena for 2 min, followed by 5 min recordings. Afterwards all fish were returned to the system. All recordings were performed between 9 and 12 am with fish fed at least 1 h prior to the experiment. For the regeneration studies, fish were preselected according to their social preference (see Supplementary Figure S1). The time spent in the social zone was calculated using the EthoVision^®^ XT software (Noldus Information Technology, version 11.5). Moreover, distance moved (in cm), velocity (in cm/s) and frequency of entering the social zone as well as heat maps representing the average location of the fish were analyzed. The experiment was performed with 18 fish for the establishment of the paradigm (Figure 2) and six fish for the regeneration time course (Figure 3). In total, 110 fish were tested for the assessment of their social behavior (Supplementary Figure S1).

### 2.5 Optokinetic Response Test

**FIGURE 4:**
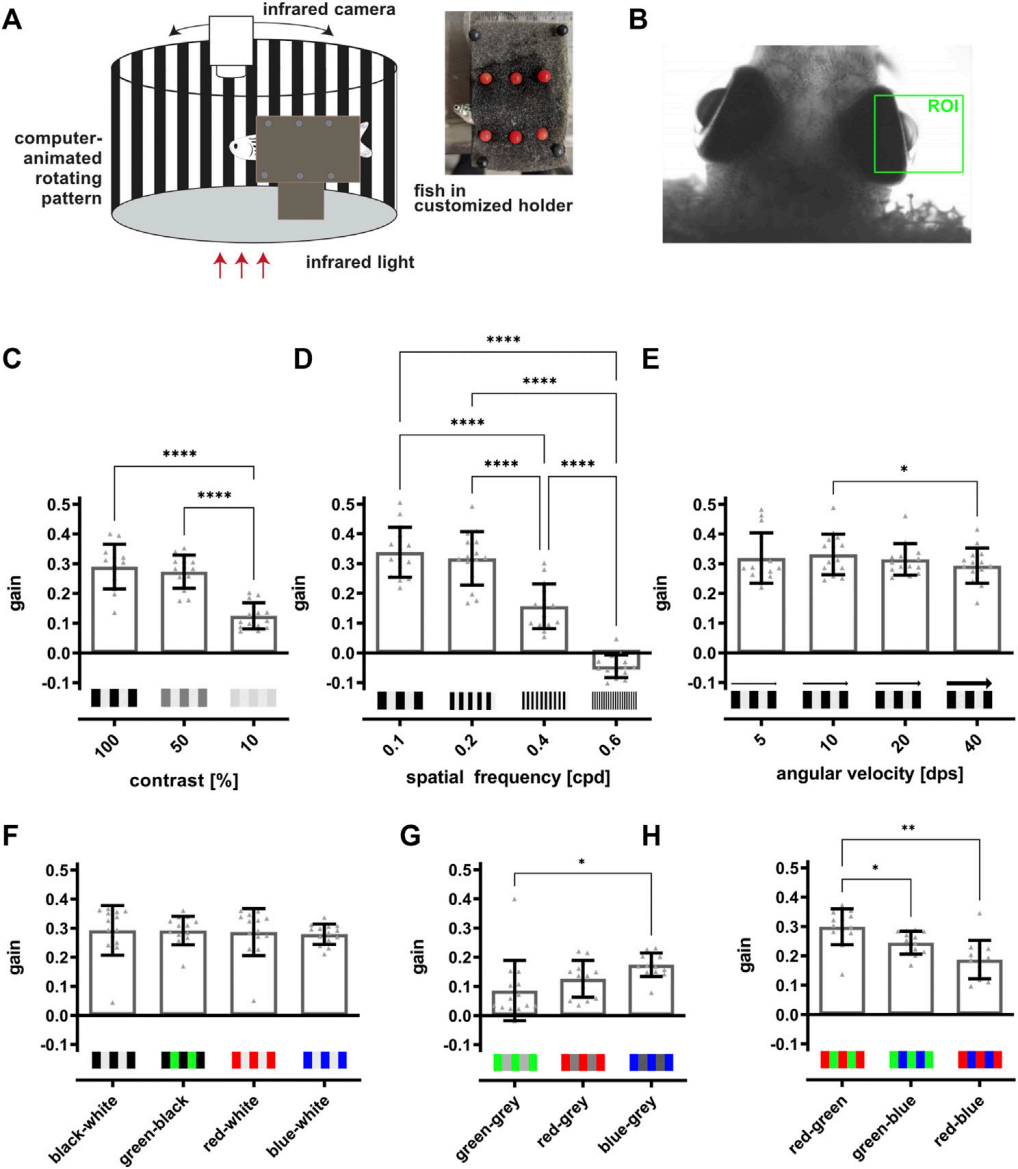
The optokinetic response of adult zebrafish is dependent on contrast, spatial frequency and color of the stimulus. **(A)** Scheme of the optokinetic response (OKR) setup. Utilizing a customized holder (inset), a test fish is placed in the middle of a round arena in which a computer-animated stimulus is projected onto a white drum surrounding the fish. The movement of the eyes is recorded from the top using an infrared-sensitive camera and an infrared light source placed beneath the fish. **(B)** Representative image of a fish placed in the OKR setup. The region of interest (ROI) for tracking the movements of the right eye is indicated in green. **(C)** Quantification of the gain obtained with 100, 50, and 10% contrast showed that the values for 100 and 50% contrast (0.29 ± 0.07 and 0.27 ± 0.05) are highly similar and are significantly increased in comparison to the value for 10% contrast (0.12 ± 0.04). **(D)** Quantification of the gain dependent on the spatial frequency revealed a significant drop from 0.1 (0.34 ± 0.08) over 0.2 (0.32 ± 0.09) and 0.4 (0.16 ± 0.07) to 0.6 cpd (−0.04 ± 0.04). **(E)** Quantification of the gain in dependence on angular velocity indicated no changes of the gain when the angular velocity was increased (5 dps: 0.32 ± 0.09, 10 dps: 0.33 ± 0.07, 20 dps: 0.31 ± 0.05, 40 dps: 0.29 ± 0.06). **(F)** Quantification showed that the gain is similar when black-white (0.29 ± 0.08), green-black (0.29 ± 0.05), red-white (0.29 ± 0.08) or blue-white stripes (0.28 ± 0.03) were used. **(G)** Matching the brightness of each grey level to the brightness of the respective color resulted in an overall lower gain compared to colored stripes on a black or white background. Blue-grey stripes elicited a significantly higher gain as compared to green-grey stripes (green-grey: 0.09 ± 0.01, red-grey: 0.13 ± 0.06, blue-grey: 0.17 ± 0.04). **(H)** Quantification revealed that two colored stimuli result in highest gains for red-green (0.3 ± 0.04), followed by green-blue (0.24 ± 0.04) and red-blue (0.19 ± 0.06). Statistics: all data are represented as mean ± SD, one-way ANOVA with Tukey’s multiple comparison test, *p* < 0.05 (*); 0.01 (**); 0.001 (***) or 0.0001 (****).

**FIGURE 5:**
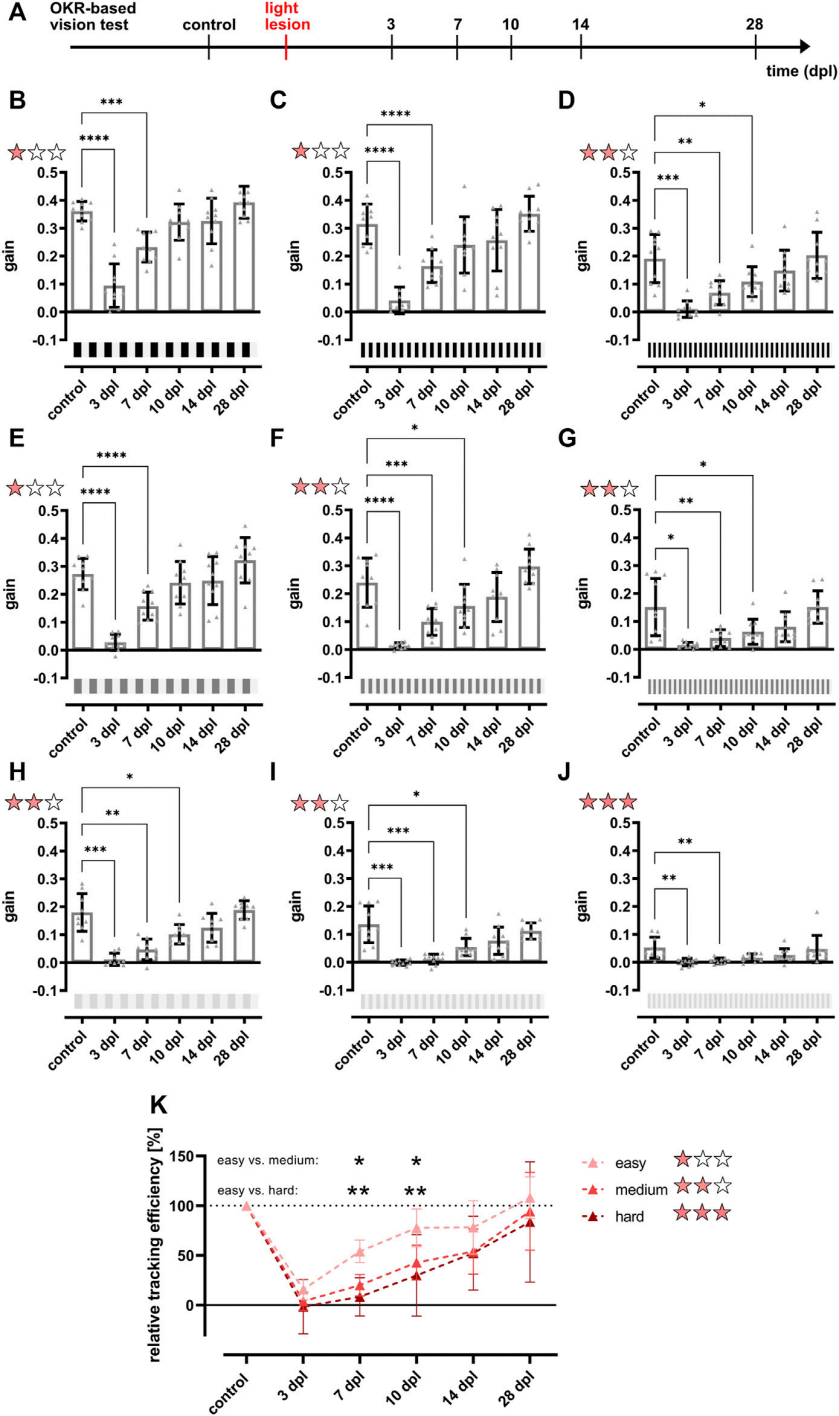
The restoration of the full optokinetic response is accomplished between 10 and 14 days after light lesion dependent on the degree of difficulty. **(A)** Experimental timeline. The optokinetic response (OKR)-based vision test was performed before the light lesion (control) and at 3, 7, 10, 14, and 28 days post lesion (dpl). **(B–J)** Quantifications of the gain obtained for a black-white stimulus at a constant angular velocity of 15 dps but different combinations of contrast and spatial frequency revealed that the gain dropped significantly at 3 dpl and remains strikingly reduced at 7 dpl at all conditions. For easy conditions with high contrast and low spatial frequency (one red star), pre-lesion levels are reached at 10 dpl, whereas the OKR for medium difficulty (two red stars) returned to pre-lesion levels from 14 dpl onwards. Under hard conditions (three red stars) with low contrast and high spatial frequency, the gain was already very low at control recordings but statistically recovered by 10 dpl. All individual data points are summarized in Table 1. **(K)** Comparison of the relative tracking efficiency of all degrees of difficulty showed a significantly higher relative tracking efficiency at 7 and 10 dpl under easy conditions compared to medium and hard conditions. Statistics: all data are represented as mean ± SD, one-way ANOVA with Dunnett’s multiple comparison test against control **(B–J)**, two-way ANOVA with Tukey’s multiple comparison test (K), *p* < 0.05 (*); 0.01 (**); 0.001 (***) or 0.0001 (****).

**FIGURE 6:**
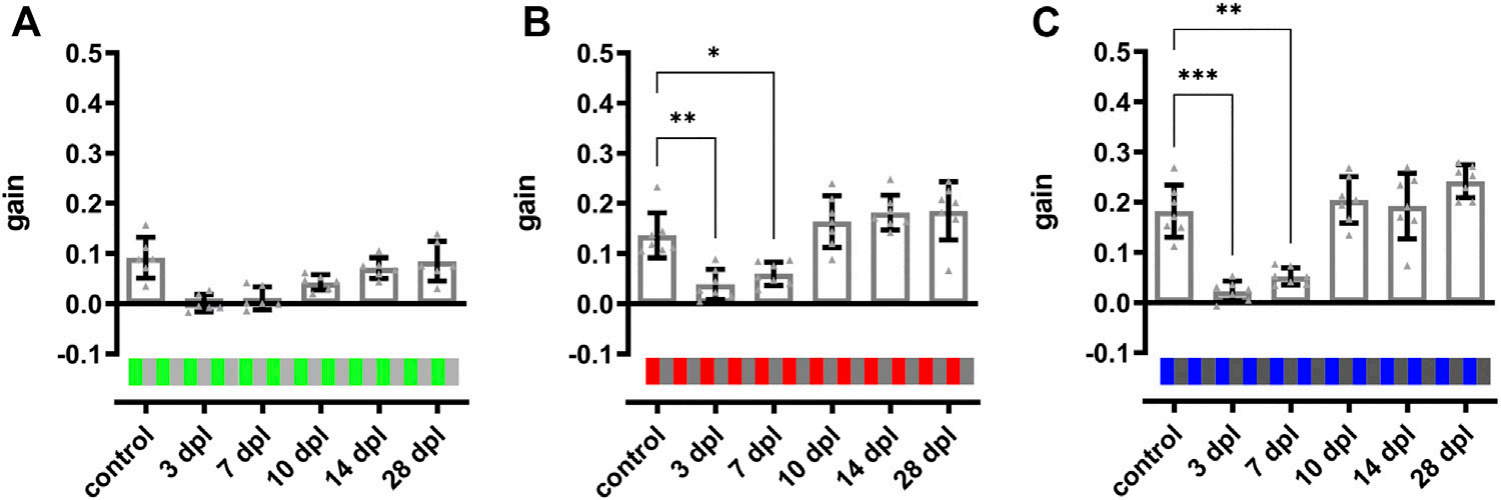
Color vision is restored at 10 days after light lesion. Quantifications of the gain obtained after light lesion for red **(B)** and blue **(C)** colored stimuli on an intensity-matched grey background (100%/0.2 cpd/15 dps) revealed a significant reduction at 3 and 7 dpl but restoration to baseline levels at 10 dpl. The quantification of the gain for a green stimulus **(A)** showed a similar trend but was not significantly different. Individual data are summarized in Table 2. Statistics: all data are represented as mean ± SD, one-way ANOVA with Dunnett’s multiple comparison test against control, *p* < 0.05 (*); 0.01 (**); 0.001 (***) or 0.0001 (****).

A modified commercial system (VisioTracker, TSE Systems GmbH) was employed to generate the stimulus and to measure the optokinetic response (OKR) of adult zebrafish as previously described (Mueller and Neuhauss, 2010; Mueller et al., 2011). Prior to the measurements, fish were briefly anaesthetized in 0.024% MS-222. Subsequently, fish were immobilized in a customized holder (Figure 4A). Therefore the anaesthetized fish was carefully placed on a foam-covered piece of Styrofoam and covered up with a thin layer of foam that was fixed with pins positioned carefully around the fish. The head and gills remained uncovered to ensure proper breathing. Fish were then placed in a tank filled with system water. The fish were surrounded by a white drum (Ø 10 cm) on which a moving stimulus generated by the TSE software was projected. Different parameters of the stimulus like contrast (in %), color, spatial frequency (in cycles per degree (cpd)) and angular velocity (in degrees per second (dps)) can be controlled using the provided software. Prior to the recordings, each fish was presented a training-stimulus (100%, black-white, 0.2 cpd, 15 dps) for up to 5 min to ensure that effects of the anesthesia had subsided and reliable tracking results could be achieved. Then, fish were presented different stimuli twice, generally going from difficult to easy followed by the reverse order. For the two-level vision test, the stimuli were presented in the following order: 0.4 to 0.3 to 0.2 cpd and reverse at 100% contrast, followed by the same order at 30 and 10% contrast. The colors for the second level were presented in the order red-blue-green and then in reverse, in three installments of varying backgrounds starting with backgrounds of equal brightness for each color (90% white-green, 60% white-blue, 20% white-red), followed by backgrounds with the highest contrast for each color (red-white, blue-white, green-black) and finally colored backgrounds (red-blue, red-green, green-blue). In total, the duration of the vision test added up to about 13 min for each fish. After the recordings, fish were freed from the holder, carefully placed into fresh system water and returned to the system. The recordings were automatically processed and analyzed in real time, inferring angular changes from eye orientation in relation to a horizontal axis to calculate eye velocity. To obtain the velocity of the tracking phase, segments of rapid movements for eye readjustments (saccades) were automatically filtered from raw measurements. Further analysis was conducted with custom made R scripts based on processed data to calculate the average gain (tracking phase velocity to stimulus velocity) for each tested condition. For testing the different stimulus parameters we assessed the OKR of at least 12 fish (Figure 4; Supplementary Figure S2) and of seven fish for the vision tests after light lesion (Figures 5, 6; Supplementary Figures S3, S4).

### 2.6 Tissue Collection and Histology

Fish were sacrificed using 0.1 M MS-222 before the corneas were carefully opened to allow better penetration of the fixative. Subsequently, heads were excised and fixed in 4% paraformaldehyde in 0.1 M Phosphate buffer (pH 7.5) overnight at 4°C. For decalcification, heads were incubated in 0.5 M EDTA in 0.1 M Phosphate buffer for 3–4 days with daily exchange of the solution. Afterwards, heads were washed with ddH_2_O before being processed in a Paraffin-Infiltration-Processor (STP 420, Zeiss) according to the following protocol: ddH_2_0: 1 min; 50% ethanol (EtOH) 5 min; 70% EtOH 10 min; 96% EtOH 25 min; 96% EtOH 2 × 20 min; 100% EtOH 2 × 20 min; xylene 2 × 20 min; paraffin 3 × 40 min/60°C; paraffin 60 min/60°C. The heads were embedded using the EG1160 Embedding Center (Leica). Semi-thin sections (2 µm) were cut using an Ultracut microtome and counterstained using hematoxylin/eosin (HE, Sigma). Paraffin embedding, sectioning and staining was performed by the CMCB Histology Facility. Imaging was performed using the ZEISS Axio Imager. Z1 provided by the CMCB Light Microscopy Facility and images were processed using Fiji. A total number of five fish per time point was analyzed.

### 2.7 Optical Coherence Tomography

**FIGURE 7:**
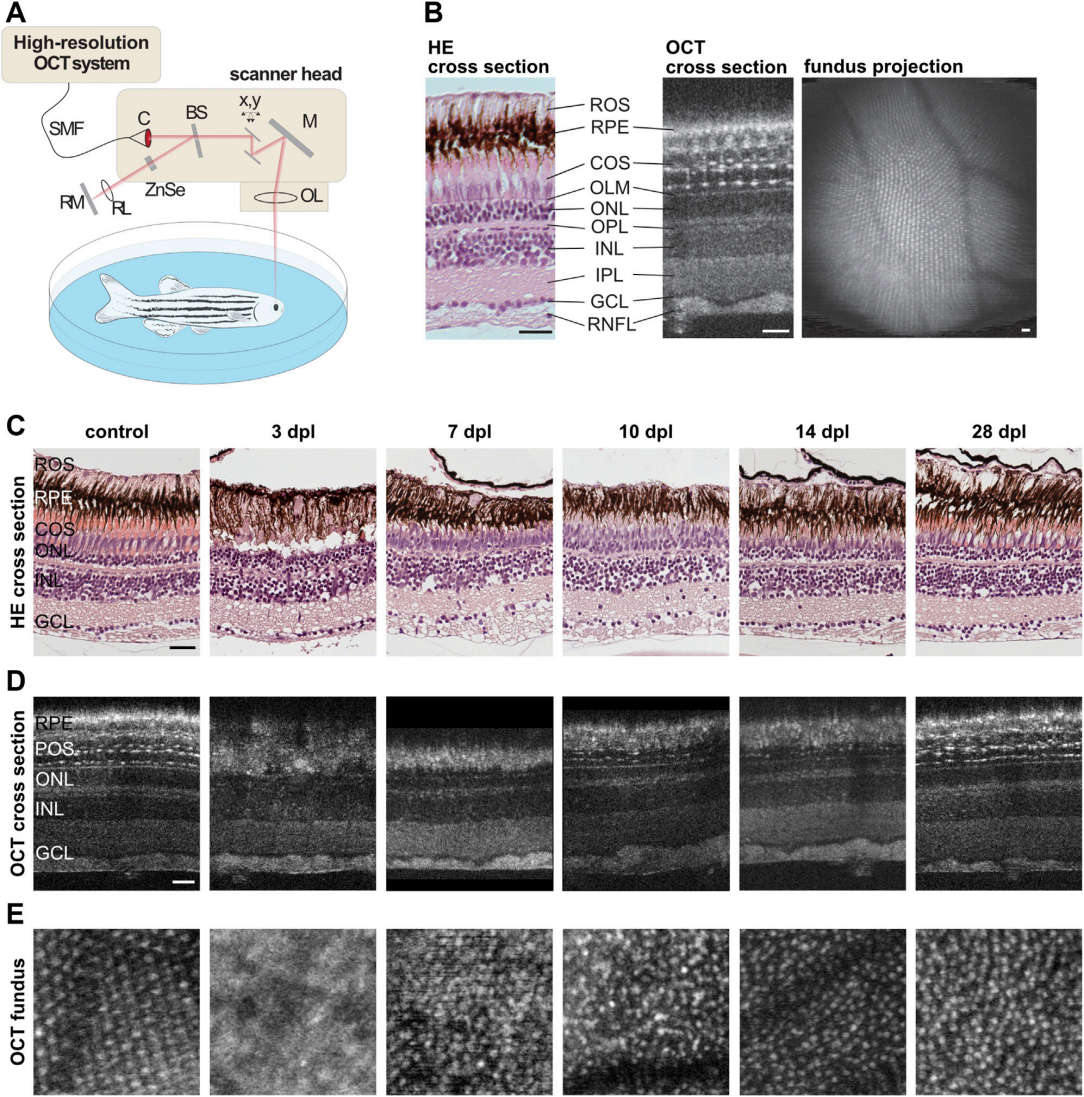
Retinal morphology is restored gradually after light lesion. **(A)** Scheme of the custom-built high resolution optical coherence tomography (OCT) setup. Laser light is guided to the scanner head via a single-mode fiber (SMF) and transferred into a free beam by a collimator (C). The incoming light is separated into a reference beam and a sample beam by a beamsplitter (BS). The sample light is deflected by two galvanometer scanners (x,y) to achieve a 2D scan pattern over the sample, reflected by a mirror (M) and focused by an adjustable focus lens (OL) into the eye where it is scattered and reflected back. The reference beam is guided through a zinc selenide glass (ZnSe) and focused by a lens (RL) before being reflected by a mirror (RM). Both beams are superimposed by the beamsplitter, spectrally resolved measured by a line scan sensor. **(B)** Comparison of a retinal cross section stained with hematoxylin/eosin (HE) and a cross section obtained via OCT imaging revealed a high degree of similarity. With the exception of the rod outer segments (ROS) which can be recognized only in HE stained samples at the top, all subsequent layers including the retinal pigment epithelium (RPE), the cone outer segments (COS), the outer limiting membrane (OLM), the outer nuclear layer (ONL), the outer plexiform layer (OPL), the inner nuclear layer (INL), the inner plexiform layer (IPL), the ganglion cell layer (GCL) and the retinal nerve fiber layer (RNFL) can be seen in both, HE and OCT. In addition, small hyper-reflective structures were observed in the photoreceptor layer imaged with OCT that mark the intersection between cone inner and outer segments. A fundus projection calculated from the OCT data showed the cone mosaic pattern as well as the retinal blood vessels in the central part of the retina. **(C)** HE stainings of unlesioned control samples as well as specimen fixed at 3, 7, 10, 14, and 28 days post lesion (dpl). **(D)** OCT cross sections of one individual fish prior to lesion and at 3, 7, 10, 14, and 28 dpl. Both methods showed the loss of photoreceptors at 3 dpl, their subsequent reappearance at 7 and 10 dpl as well as their proper differentiation at 14 and 28 dpl. **(E)** OCT fundus projections revealed the loss of the cone mosaic at 3 dpl and its subsequent recovery, although the mosaic appeared irregular even at 28 dpl. Scales represent 25 µm.

A custom-built spectrometer-based OCT setup (Figure 7A) was used for non-invasive *in vivo* imaging of the adult zebrafish retina using a supercontinuum light source (SuperK Extreme, NKT Photonics), a spectrometer with a center-wavelength of 720 nm and a bandwidth of 410 nm enabling an axial and lateral resolution of 1.3 and 7 µm in air, respectively. Yet, overall optical power input was below energy levels causing any tissue damage or phototoxicity effects during full measurements (<1 min per eye), ensuring animal welfare even in repeated measurements. In brief, laser light is guided to the scanner head via a single-mode fiber and transferred into a free beam by a collimator. The incoming light is separated into a reference beam and a sample beam by a beamsplitter. The sample light is deflected by two galvanometer scanners to achieve a 2D scan pattern over the sample. The light is then reflected by a mirror and focused by an adjustable focus lens into the eye where it is scattered and reflected back in different tissue depth. The light of the reference beam is guided through a zinc selenide plate to match the length and dispersion of the reference beam path to the corresponding values of the sample beam path including the fish eye. It is likewise focused by a lens and back reflected by a mirror. The light reflected back from the reference and sample beam are superimposed by the beam splitter and guided back to the OCT system where the light is spectrally resolved by a diffraction grating and measured by a line scan sensor. After background correction, spectral shaping and resampling to equidistant points in wavenumber the depth dependent information is calculated using a Fast-Fourier Transformation (A-scan) (Köttig et al., 2012). OCT recordings were performed as described previously (Weber et al., 2013). In brief, fish were anaesthetized in 0.02% MS-222 in fish water before being transferred into a small petri dish with 0.02–0.04% MS-222. Fish were positioned with one eye facing upwards with their heads stabilized using a small piece of foam and covered with a cover slip. In order to image approximately the same retinal area during consecutive measurements, fish were consistently oriented to align the fast-scanning axis along the dorsal-ventral axis of the eye and the slow-scanning axis with the anterior-posterior axis of the eye. An adjustable focus unit allowed to focus specifically onto the photoreceptor layer. Imaging speed was set to an A-scan rate of 12.8 kHz, which corresponds to 15 cross sectional scans per second with 800 axial scans per cross section. Finally, a three-dimensional image stack consisting of 800 cross-sections was recorded from the eye background of each animal with an image field of 0.7 × 0.7 mm^2^. After measurements, fish were transferred back into their respective cages to recover from anesthesia. Due to the curvature of the retina, OCT also uses a fan-shaped scan for image acquisition. The coordinates can be transformed with a MatLab script so that a planar representation of the mosaic structures can be recalculated from the fan-shaped OCT images. The retinal structures were then visualized using Fiji (Schindelin et al., 2012). In total, we followed the morphological recovery of five individual fish.

### 2.8 Quantification and Statistical Analysis

For statistical analysis the GraphPad prism software (version 9.0.0) was used to determine *p*-values with either unpaired t-test (Figures 1A–C; Supplementary Figures S1C, S2), one-way ANOVA with Tukey’s multiple comparison test for post hoc analysis (Figures 2, 4, 5K; Supplementary Figure S1A) or Dunnett’s multiple comparison test for post hoc analysis against control values (all remaining graphs). Chosen significance levels were ****p ≤ 0.0001; ***p ≤ 0.001; **p ≤ 0.01; *p ≤ 0.05. Values above *p* > 0.05 were not considered significant. Graphs are shown as scatter plots including mean ± SD.

## 3 Results

### 3.1 Restoration of Vision After Light Lesion is Undetectable Using a Dark-Light Preference Test in Adult Zebrafish

To date, various slightly different dark-light preference behavior setups have been used to assess visual function in larval and adult zebrafish (Serra et al., 1999; Gerlai et al., 2000; Blaser and Peñalosa, 2011; Magno et al., 2015; Facciol et al., 2019). To be able to analyze functional recovery after light lesion, we established a dark-light preference test following the general design. To this aim, we used a customized arena divided into ten individual compartments (10 × 20 cm) each made out of infrared-transparent material. Half of the compartment was composed of clear and the other half of black plastic to obtain a light and dark zone, respectively (Figure 1A). The arena was either put in a dark environment (light off) or illuminated with white light (85 lux) from the top (light on). Infrared illumination was provided from the bottom and an infrared-sensitive camera centered above the arena was used to record the movement of a single test fish. For quantification, we calculated the percentage of time the fish spent in the light zone, the distance moved and the velocity. We found that unlesioned wildtype fish spent approx. half of the time (53.1 ± 16.1%) in the light zone when the light is off (Figure 1B). In contrast, the time spent in the light zone decreased to 32.9 ± 22.7% when the light was on. Importantly, although the difference was statistically significant, the variance between individual fish was rather high. Corresponding heat maps show that fish tended to swim close to the borders of the compartment in the light off condition whereas they also explored the central parts of the compartment when the light was on (data not shown). With respect to distance moved as well as velocity, we found that fish swam significantly more with a significantly higher velocity (Figures 1C,D) in light off conditions. To analyze changes in dark-light preference due to the effect of a light lesion as well as during subsequent functional recovery, we performed light lesions after the recording under control conditions and tested the dark-light preference at 1, 3, 7, 14, and 28 days post lesion (dpl) (Figure 1E). However, in comparison to control measurements (38.9 ± 23.4%) no significant changes could be observed with respect to the time spent in the light zone neither immediately following the light lesion (1 dpl: 41.9 ± 22.1%) nor during the course of regeneration (3 dpl: 41.8 ± 24.9%, 7 dpl: 39.3 ± 22.2%, 14 dpl: 43.6 ± 20.9%, 28 dpl: 56.3 ± 16.6%) (Figure 1F). Similarly, distance moved as well as velocity did not change significantly following a light lesion and subsequent regeneration (Figures 1G,H). Again, strong variations were observed between individual fish. Taken together, our results show that dark-light preference reveals significant differences under light and dark conditions in unlesioned fish but that individual variability impedes assessment of functional recovery after light lesion.

### 3.2 A Vision-Dependent Social Preference Test Allows the Assessment of Visual Function Following Light Lesion in Adult Zebrafish

To assess visual function in a more sensitive manner, we established a vision-dependent social preference test for adult zebrafish making use of their inherent preference to socialize (Engeszer et al., 2007; Suriyampola et al., 2016; Orger and De Polavieja, 2017). To this end, a customized T-shaped arena was divided into two completely separated compartments (Figure 2A). The lower arm harbored a group of fish representing the social stimulus whereas the upper arm (10 × 50 cm = 500 cm^2^) contained a single test fish. A transparent window represented the only non-opaque interface between the two compartments and the zone in front of the window was defined as social zone (red box, 5 × 9 cm = 45 cm^2^). Movement of the test fish was recorded using an infrared-sensitive camera above the arena which was illuminated by an infrared light source placed beneath the fish. To demonstrate visual dependence of the setup, we performed recordings of test fish under normal light conditions (light on) with and without conspecifics as a social stimulus as well as in the dark (light off) with a social stimulus. The respective recordings (light off with social stimulus, light on without social stimulus, light on with social stimulus) were performed on three subsequent days. Without illumination, test fish did not spend much time in the social zone and stayed in close proximity to the borders of their compartment even in presence of a social stimulus as indicated by representative heat maps (Figure 2B). Similar results were observed if the arena was illuminated but no social stimulus was present, although test fish also explored the more central parts of the compartment. In stark contrast, test fish stayed mainly in the social zone and only rarely explored other parts of the compartment when illumination was on and a social stimulus was present. Importantly, we observed that the preference to respond to the social stimulus was highly variable from individual to individual and that some fish did not change their behavior in correlation to the presence of the social stimulus (Supplementary Figure S1A). Hence, we analyzed a larger group of fish to address this variability. In total, fish spent almost 40% (35.5 ± 24.9%) of their time in the social zone with values from 0 to almost 100% (Supplementary Figure S1B). Based on these data as well as on general swimming behavior, we subdivided fish into “social” and “unsocial” animals. In essence, fish spending considerably more time within the social zone in the presence of a social stimulus and displaying a normal swimming behavior were selected while all others were dismissed. To test if the preference to socialize is indeed a stable trait, we re-examined the “social” fish 7 days after the initial recordings and found no statistically significant differences (Supplementary Figure S1C). Subsequently, all further experiments were conducted with preselected “social” fish. Quantification of the time spent in the social zone confirmed the results of the heat maps (Figure 2C). Test fish spent on average almost 60% (59.3 ± 17.9%) of their time in the social zone when presented a social stimulus which drops significantly to 17.1 ± 10.6% under light off conditions and to 5.3 ± 3.7% in the absence of a social stimulus. We also quantified distance moved, velocity and frequency of entering the social zone. We found that fish swam significantly more in the absence of a social stimulus compared to other conditions (Figure 2D). Similarly, fish displayed a significantly higher velocity in the absence of a social stimulus in comparison to dark condition which further dropped in the presence of a social stimulus (Figure 2E). Finally, the frequency of entering the social zone was significantly reduced in the absence of a social stimulus compared to the other conditions (Figure 2F). In summary, these data show that our social preference test allows addressing behavior that is completely dependent on vision.

### 3.3 Vision is Rapidly Lost After Light Lesion but Restored Within 7 days

To examine potential changes of visual performance in the context of regeneration, we made use of our social preference test. Initially, unlesioned controls were recorded in an illuminated arena with a group of conspecifics as social stimulus. Subsequently, a light lesion was applied to the same animals and their performance in the social preference test was recorded at 1, 3, 7, 14, and 28 dpl (Figure 3A). Heat maps of one individual representative test fish indicating the mean position of the fish during each recording are shown in Figure 3B. Prior to the lesion, the fish spent the majority of time within the social zone. However, at 1 and 3 dpl the fish displayed a completely different behavior and spent most of the time outside of the zone exploring the whole compartment although staying close to its borders. The fish started to spend more time within the social zone at 7 dpl and returned to initial levels from 14 dpl onwards. Quantifications confirmed the significant drop of the time the fish spent in the social zone at 1 and 3 dpl (4.6 ± 2%, 10.8 ± 8.8%) compared to control (34.5 ± 8.4%) (Figure 3C). Statistically, no difference was observed at 7 dpl compared to controls (23.8 ± 14% versus 34.5 ± 8.4%). However, full recovery for all animals was only reached from 14 dpl onwards (14 dpl: 34.8 ± 8.7%, 28 dpl: 34.1 ± 4.7%). No significant changes were seen in the distance moved, velocity and frequency of entering the zone (Figures 3D–F). Based on these observations, we concluded that visual function is rapidly and significantly impaired due to the light lesion but that vision (at least to some extent) is restored already within 7 days after lesion.

### 3.4 The Optokinetic Response of Adult Zebrafish Depends on Contrast, Spatial Frequency and Color of the Stimulus

Our results of the social preference test indicate that visual performance to recognize basal traits is recovered within a short timeframe after light lesion. However, to analyze visual function in more depth, we turned to the optokinetic response (OKR) assay. OKR is a reflexive behavior present in all vertebrates and improves the animal’s ability to track moving objects or stimuli. This is achieved by stabilizing the image of the object on the retina by matching the direction and velocity of the movement of the eyes to the movement of the stimulus (Neuhauss, 2003). To analyze the OKR in adult zebrafish, we used a modified VisioTracker setup (TSE Systems GmbH) (Figure 4A). In detail, fish were carefully secured in a custom-made holder between two pieces of foam, keeping the head and the gills uncovered. The immobilized fish was then put into a water-filled tank in an upright position and recorded from the top using an infrared-sensitive camera and infrared illumination from below. A computer-generated stimulus was projected onto a white drum surrounding the fish. In the company-provided software we defined a region of interest (ROI) for each eye to allow the tracking of movements of both eyes individually (Figure 4B). To elicit the OKR, a stimulus composed of black-white or differently colored vertical stripes was presented to the fish. In addition, the stimulus was modified with respect to contrast, number (spatial frequency) and speed (angular velocity) of the stripes. To quantify the response, we used the gain which is defined as the ratio of the tracking speed of the eyes to the speed of the stimulus. In a first set of experiments, we used black-white stripes and modified either contrast (in %), spatial frequency (in cycles per degree (cpd)) or angular velocity (in degrees per second (dps)) while keeping the other two parameters constant. A decrease in contrast from 100 to 50 and finally 10% with constant spatial frequency (0.2 cpd) as well as constant angular velocity (15 dps) resulted in a similar gain for 100% (0.29 ± 0.07) and 50% (0.27 ± 0.05) but a significantly decreased gain for 10% (0.12 ± 0.04) (Figure 4C). Increasing the spatial frequency from 0.1 to 0.2 to 0.4 and finally to 0.6 cpd with constant contrast (100%) as well as constant angular velocity (15 dps) also resulted in a highly significant reduction of the obtained gain (Figure 4D). While spatial frequency of 0.1 and 0.2 cpd resulted in a similar gain (0.34 ± 0.08 and 0.32 ± 0.09), the gain was reduced by 50% with a spatial frequency of 0.4 cpd (0.16 ± 0.07) and even yielded a negative value with a spatial frequency of 0.6 cpd (−0.04 ± 0.04) indicating that the fish were not able to track the stimulus. Modification of the angular velocity from 5 to 10 to 20 and finally to 40 dps with constant contrast (100%) as well as constant spatial frequency (0.2 cpd) had no major consequences on the obtained gain, apart from a statistically significant, but minor reduction from 10 to 40 dps (5 dps: 0.32 ± 0.09, 10 dps: 0.33 ± 0.07, 20 dps: 0.31 ± 0.05, 40 dps: 0.29 ± 0.06) (Figure 4E). In a second set of experiments, we addressed the ability of the fish to perceive colors. To this aim, we modified the color of the stripes (green, blue, red) but kept contrast (100%), spatial frequency (0.2 cpd) and angular velocity (15 dps) constant. First, we used colored stripes on either black or white background (green-black, red-white, blue-white) in comparison to black-white stripes (Figure 4F). We found that the gain for black-white (0.29 ± 0.08), green-black (0.29 ± 0.05) red-white (0.29 ± 0.08) and blue-white (0.28 ± 0.03) were highly similar with no significant differences. However, during our experiments, we also tested the complementary green-white, red-black and blue-black stripe combination and noticed that the background color has a substantial influence on the resulting gain (Supplementary Figure S2). For instance, green-white stripes resulted in a significantly lower gain in comparison to green-black. In contrast, red and blue stripes resulted in a lower gain when combined with black stripes in comparison to their combination with white stripes. These results indicate that the fish did not only perceive color but also changes in contrast under these experimental conditions. Hence, to minimize the influence of contrast differences between color and background, we next generated a stimulus with the individual color combined with a brightness-matched grey background. For this, we measured the brightness of the green-only, red-only and blue-only stimulus and matched the grey value of the background accordingly (Figure 4G). We found that the gain is reduced from blue-grey (0.17 ± 0.04) over red-grey (0.13 ± 0.06) to green-grey (0.09 ± 0.01), although only the difference between blue-grey and green-grey was significantly different. As expected, we observed an overall decline in the gain compared to color on black or white background. Finally, we tested the visual performance using colored stripes on colored background. We found that a red-green stimulus elicited the highest gain (0.3 ± 0.04), followed by a green-blue (0.24 ± 0.04) and red-blue (0.19 ± 0.06) stimulus (Figure 4H). In essence, our results show that color, contrast and spatial frequency but not angular velocity of the stimulus have an influence on the OKR in adult zebrafish.

### 3.5 An OKR-Based Two Level Vision Test Reveals Gradual Recovery of Visual Properties Within 10–14 days After Light Lesion

After establishment of the OKR setup, we developed a two level vision test for adult zebrafish that allows the thorough assessment of visual function similar to a human vision test. In the first level, contrast sensitivity and spatial resolution were tested using a black-white stimulus with variations in contrast (100, 30, and 10%) and spatial frequency (0.2, 0.3, and 0.4 cpd) but a constant angular velocity (15 dps) yielding nine different combinations of stimuli. In the second level, the ability to perceive colors was assessed using a colored stimulus on an intensity-matched grey background keeping contrast (100%), spatial frequency (0.2 cpd) and angular velocity (15 dps) constant. To analyze functional recovery after light lesion, we determined the OKR of the test fish prior to the lesion (control) and at 3, 7, 10, 14, and 28 dpl (Figure 5A). All individual data points are summarized in Table 1. To simplify the subsequent analysis, the nine possible combinations of the first level of the vision test were divided into three degrees of difficulty depending on the obtained gain of the control recordings (Figures 5B–J). A gain greater than 0.25 was considered an easy condition (indicated with one red star) which comprised three stimuli combinations with 100% contrast and a spatial frequency of 0.2 cpd, 100% contrast and a spatial frequency of 0.3 cpd as well as 30% contrast and a spatial frequency of 0.2 cpd (Figures 5B,C,E). Five conditions with a gain between 0.1 and 0.25 were defined as medium (two red stars) comprising the conditions with 100% contrast and a spatial frequency of 0.4 cpd, 30% contrast and a spatial frequency of 0.3 and 0.4 cpd as well as 10% contrast and a spatial frequency of 0.2 and 0.3 cpd, respectively (Figures 5D,F–I). One condition with 10% contrast and a spatial frequency of 0.4 cpd resulted in a gain below 0.1 and was defined as hard (three red stars, Figure 5J). Next, we performed OKR measurements of the described regime over the course of regeneration. We found that the gain was significantly reduced at 3 dpl for all tested conditions indicating that vision is severely impaired. At 7 dpl, the gain increased for all conditions except for the hard condition with 10% contrast and a spatial frequency of 0.4 cpd which remained unchanged. However, despite the increase in gain, all values were still significantly different from pre-lesion levels for all conditions. At 10 dpl, a further increase in gain was observed in all conditions and all three easy conditions were no longer statistically different from pre-lesion levels indicating recovery of visual function to a certain degree. Nevertheless, despite the absence of a statistical difference, easy conditions showed further improvements until 28 dpl when contrast sensitivity and spatial resolution were fully restored to pre-lesion levels. In addition to the easy conditions, the gain of the hard condition also showed no statistical difference from 10 dpl as well. Admittedly, this finding is most likely due to the experimental setup which yielded only a gain below 0.1 already at pre-lesion levels rather than reflecting a full recovery of vision. At 14 dpl, all conditions showed a further increase in gain and all five medium conditions were no longer statistically different from pre-lesion levels indicating improved vision. Again, even though not statistically different, full pre-lesion levels were only reached at 28 dpl. For better comparison, we combined the obtained data points of the three degrees of difficulty. To account for differences between individual fish, we normalized the data of each day and condition after the lesion with respect to the control gain. Subsequently, we calculated the “relative tracking efficiency” by averaging our normalized values for each time point according to the respective degree of difficulty. All individual data are summarized in Supplementary Table S1. For easy conditions, the relative tracking efficiency was reduced significantly at 3 and 7 dpl, but no significant difference to control could be observed from 10 dpl (Supplementary Figure S3A). However, pre-lesion levels were only restored at 28 dpl. With respect to the medium degree of difficulty, a highly significant drop in the relative tracking efficiency was observed early after lesion which was only resolved by 28 dpl (Supplementary Figure S3B). For the hard degree of difficulty, the decline of the visual performance was reversed by 14 dpl (Supplementary Figure S3C). The direct comparison between the three degrees of difficulty revealed a significantly higher relative tracking efficiency for easy conditions compared to both medium and hard at 7 and 10 dpl (Figure 5K). Taken together, our data show a striking correlation between the quality and kinetics of visual recovery and the degree of difficulty of the visual task. In general, easier tasks that require less contrast sensitivity and spatial resolution are recovered faster and to a higher degree of quality. In contrast, more demanding tasks that require improved visual performance take comparably longer time to recover. In the second level of the vision test, we chose a green, red or blue stimulus on an intensity-matched grey background. Starting from an already low gain obtained at pre-lesion levels for the green stimulus, a statistically non-significant drop was observed at 3 and 7 dpl which increased at 10 and 14 dpl before returning to control levels at 28 dpl (Figure 6A). Similarly, the gain obtained at pre-lesion levels from a red or blue stimulus was significantly reduced at 3 and 7 dpl but returned to pre-lesion levels already at 10 dpl (Figures 6B,C). We also determined the gain for various colors on a black or white background as well as colored stripes on colored backgrounds. All show a significant drop in gain at 3 and 7 dpl and return to pre-lesion levels at 10 dpl (Supplementary Figure S4; Supplementary Table S2). Taken together, these data show that contrast sensitivity, spatial resolution and color perception are gradually but fully recovered after light lesion.

**TABLE 1:** Individual data points for the first level of the OKR-based vision test in the course of regeneration.

OKR parameters				Gain					
Color	Contrast [%]	SF [cpd]	AV [dps]	Control	3 dpl	7 dpl	10 dpl	14 dpl	28 dpl
black-white	100	0.2	15	0.35 ± 0.04	0.09 ± 0.07	0.23 ± 0.05	0.32 ± 0.06	0.37 ± 0.04	0.42 ± 0.08
black-white	100	0.3	15	0.3 ± 0.07	0.04 ± 0.04	0.17 ± 0.05	0.25 ± 0.1	0.32 ± 0.05	0.41 ± 0.05
black-white	100	0.4	15	0.18 ± 0.08	0.01 ± 0.03	0.07 ± 0.04	0.11 ± 0.05	0.19 ± 0.07	0.29 ± 0.08
black-white	30	0.2	15	0.27 ± 0.06	0.03 ± 0.03	0.16 ± 0.04	0.24 ± 0.07	0.29 ± 0.04	0.38 ± 0.05
black-white	30	0.3	15	0.22 ± 0.09	0.01 ± 0.01	0.1 ± 0.04	0.16 ± 0.07	0.25 ± 0.05	0.35 ± 0.06
black-white	30	0.4	15	0.13 ± 0.1	0.01 ± 0.01	0.04 ± 0.03	0.06 ± 0.04	0.13 ± 0.05	0.2 ± 0.06
black-white	10	0.2	15	0.17 ± 0.07	0.01 ± 0.02	0.05 ± 0.04	0.11 ± 0.04	0.2 ± 0.04	0.19 ± 0.05
black-white	10	0.3	15	0.12 ± 0.07	0.0 ± 0.01	0.01 ± 0.02	0.05 ± 0.03	0.13 ± 0.04	0.11 ± 0.03
black-white	10	0.4	15	0.04 ± 0.03	0.0 ± 0.01	0.0 ± 0.01	0.02 ± 0.01	0.04 ± 0.03	0.03 ± 0.01

Abbreviations: AV, angular velocity; cpd, cycles per degree; dpl, days post lesion; dps, degrees per second; SF, spatial frequency.

**TABLE 2:** Individual data points for the second level of the OKR-based vision test during the course of regeneration.

OKR parameters				Gain					
Color	Contrast [%]	SF [cpd]	AV [dps]	Control	3 dpl	7 dpl	10 dpl	14 dpl	28 dpl
green-grey	100	0.2	15	0.09 ± 0.04	0.0 ± 0.01	0.0 ± 0.02	0.04 ± 0.01	0.06 ± 0.0	0.08 ± 0.0
red-grey	100	0.3	15	0.14 ± 0.04	0.04 ± 0.03	0.06 ± 0.03	0.16 ± 0.05	0.17 ± 0.01	0.16 ± 0.09
blue-grey	100	0.4	15	0.18 ± 0.05	0.02 ± 0.02	0.05 ± 0.02	0.2 ± 0.04	0.18 ± 0.01	0.23 ± 0.03

AV, angular velocity; cpd, cycles per degree; dpl, days post lesion; dps, degrees per second; SF, spatial frequency.

### 3.6 The Photoreceptor Layer is Restored Morphologically Within 14 days After Light Lesion

Our behavioral tests indicate that functional vision is gradually restored between 7 and 14 dpl. Hence, we were interested in the underlying morphological processes that occur in the same timeframe. To this aim, we made use of two different techniques to assess morphological regeneration: classical histological hematoxylin and eosin staining (HE), as well as optical coherence tomography (OCT). OCT is a non-invasive imaging technique which allows the repeated analysis of retinal structures *in vivo* (Walther et al., 2011). To address degeneration and regeneration in the zebrafish retina following a light lesion, we established a custom-built spectral domain-OCT system that was specifically adapted to the anatomy of the zebrafish eye (Figure 7A) (Gaertner et al., 2014). Direct comparison of a retinal cross section stained with HE and a cross section obtained *via* OCT revealed a high degree of similarity (Figure 7B). Both methods allow the discrimination of all different retinal layers. In this context, nuclear layers appear purple in HE stainings and dark in OCT images whereas plexiform layers appear light pink in HE stainings and brighter in OCT images, respectively. Interestingly, OCT revealed small hyper-reflective structures that are regularly aligned within the photoreceptor layer which have been assigned to the inner-outer segment junctions of photoreceptors allowing the examination of the photoreceptor fine structure (Fernández et al., 2001). In addition to the resolution of retinal layers in great detail, OCT allows the acquisition of fundus images showing the mosaic cone pattern as well as retinal blood vessels (Figure 7B). To obtain insights into the regeneration of photoreceptors after light lesion, we employed HE stainings and OCT imaging in parallel. For HE, we used fixed specimen of unlesioned controls as well as light lesioned animals sacrificed at 3, 7, 10, 14, and 28 dpl (Figure 7C). We observed that all retinal layers were clearly distinguishable and well organized prior to the lesion. In particular, the outer segments of the photoreceptors were well defined. In stark contrast, the photoreceptor layer was highly disordered and displayed massive cell loss and disruptions at 3 dpl whereas the other layers remain largely unaffected by the lesion. Outer segments of photoreceptors cannot be recognized at this time point within the lesioned area. At 7 dpl, the photoreceptor layer reappeared and continued to grow in size until 14 dpl. Concomitantly with the reappearing of the photoreceptor layer, the outer segments grew in size and became more organized. At 28 dpl, the retinal architecture is largely restored and highly similar to unlesioned controls. Using OCT imaging, we followed individual fish over a period of 28 days after the lesion (Figure 7D). The corresponding OCT cross sections confirmed the results from the HE staining with respect to the destruction and recovery of the photoreceptor layer after light lesion. In addition, the hyper-reflective spots in the photoreceptor layer that were lost at 3 and 7 dpl, reappeared at 10 dpl and became gradually more organized until 28 dpl. However, even at this latest stage, their distribution appeared less organized in comparison to controls. We further analyzed the fundus projections to monitor the cone mosaic (Figure 7E). We found that photoreceptors were clearly arranged in a stereotypic mosaic in unlesioned controls. In contrast, the mosaic could not be distinguished at all at 3 dpl. Starting from 7 dpl onwards, cone cells reappeared, however, the mosaic did not regain its clear and well-organized structure observed prior to lesion, not even at 28 dpl. In conclusion, our data show that morphological restoration occurs gradually within a 14 day timeframe upon light lesion.

## 4 Discussion

Regeneration is the natural process of replacing and restoring damaged or missing cells, tissues, organs or even entire body parts to full function (Goss, 1969). Specifically, regeneration in the central nervous system is only considered complete with both structural and functional reintegration (Carlson, 2007). However, most studies on retina regeneration in zebrafish mainly focus on morphological criteria to assess the outcome of regeneration (Angueyra and Kindt, 2018). The need to study functional integration of newly regenerated cells into the existing circuity is also heavily debated in the field (Fleisch et al., 2011; Angueyra and Kindt, 2018; Lahne et al., 2021; Stella et al., 2021). Functional regeneration can be addressed on several levels. On the cellular level, dendritic complexity and synaptic function have been studied by immunohistochemistry whereas electroretinography allows studying the functional output on whole tissue level (Mensinger and Powers, 2007; McGinn et al., 2018, McGinn et al., 2019). Behavior, as the ultimate output of neural activity, provides insights on the organismal level and several established vision-dependent assays allow the evaluation of visual performance in zebrafish (Neuhauss, 2003; Mueller and Neuhauss, 2010). Furthermore, some behavioral studies have analyzed visually-controlled behavior in a regeneration dependent context and implicate that vision is functionally restored after retinal lesion (Lindsey and Powers, 2007; Sherpa et al., 2008; Maurer et al., 2014; Sherpa et al., 2014; Wang et al., 2017). However, in depth studies addressing specific parameters of vision, e.g. contrast sensitivity or spatial resolution, are currently lacking. Here, we investigated different vision-dependent behavioral tests to study specifically the kinetics and the level of visual recovery following light-mediated ablation of photoreceptors.

Using a dark-light preference test that has been frequently used to address anxiety behavior or drug-related changes (Blaser and Peñalosa, 2011; Facciol et al., 2017; Magno et al., 2015; Serra et al., 1999), we observed a slight preference for the dark zone over the light zone in unlesioned fish. However, we failed to monitor lesion-dependent changes because of a high interindividual variability which is consistent with previous observations (Figure 1) (Demin et al., 2019; Volgin et al., 2019). In addition, our applied lesion paradigm does not result in complete blindness because it spares photoreceptors at the ventral and very dorsal parts of the retina (Weber et al., 2013). Hence, dark-light conditions in our setup might still be recognized even after light lesion explaining the lack of lesion-dependent behavioral changes. Interestingly, Wang and colleagues reported alterations in the dark-light preference early after light-induced lesion (Wang et al., 2017). However, they also reported a fast recovery already at 3 dpl and a general decrease of movement after lesion, indicating that this finding might also be assigned to secondary effects of the lesion. As the dark-light preference test was found unsuitable to assess visual performance in our hands, we made use of the natural preference of zebrafish to stay close to their conspecifics (Engeszer et al., 2007; Orger and De Polavieja, 2017; Suriyampola et al., 2016). Place preference tests that assess the time fish spend in close proximity to a social stimulus have already been reported (Ogi et al., 2021). However, they were used mainly to address specific aspects of social behavior, anxiety-related behaviors or drug-related behavioral changes (Dreosti et al., 2015; Varga et al., 2018) but not in the context of vision and retinal regeneration. We found that unlesioned fish spent only little time in the social zone in the dark (light off) or in the absence of a social stimulus (Figure 2). In stark contrast, fish spend the majority of their time in the social zone if presented a social stimulus. This indicates that our social preference test is highly or even solely dependent on vision because the stimulus perception is only allowed through a clear window connecting the two completely separated compartments. We hence concluded that our setup is well suited for the assessment of the visual performance of individual test fish. In the subsequent regeneration context, we found that the social preference behavior is initially drastically impaired but restored to pre-lesion levels by 7 dpl (Figure 3). These data show that visual performance is reestablished rapidly upon light lesion. However, basic recovery of vision is most likely already sufficient to react to the presented social stimulus because visual requirements for zebrafish to socialize are comparably low and are only determined by biological motion and conspecific form (Nunes et al., 2020). To address visual performance on a higher level of detail, we established a vision test for adult zebrafish based on the optokinetic response (OKR). In comparison to other behavioral assays that are influenced by a number of endogenous and exogenous factors, OKR is a reflexive and very robust behavior (Blaser and Peñalosa, 2011; Facciol et al., 2019; Genario et al., 2020; Robert Gerlai, 2019; Gorissen et al., 2015; Mueller and Neuhauss, 2010; Van Den Bos et al., 2017). It is triggered by moving objects across the visual field and evokes stereotyped eye movements which are already established in zebrafish at 5 days post fertilization (Mueller and Neuhauss, 2010; Neuhauss, 2003). In our setup for adult animals, the OKR is highly dependent on contrast, spatial frequency and color of the stimulus (Figure 4; Supplementary Figure S2). The lowest spatial frequency that could reliably be resolved was 0.4 cpd which corresponds to an estimated visual acuity of 0.6 and is in line with other studies (Tappeiner et al., 2012). With respect to contrast sensitivity, we found that the lowest possible contrast of 10% was still tracked well by the test fish albeit with lower efficiency. Similarly to other studies, modification of the angular velocity did not result in changes of the gain (Tappeiner et al., 2012). Hence, we addressed only contrast sensitivity and spatial resolution in our study. In addition, we analyzed the ability to perceive colors because cones are the major cell type affected by our light lesion paradigm (Weber et al., 2013). We found that the tracking efficiency of the respective color is influenced by the chosen background and that the gains were lower with intensity matched backgrounds compared to black or white background. This implies that pairing colors with individually matched backgrounds provides a better readout for the perception of colors. Interestingly, blue and red colors were tracked with higher efficiency compared to green. Although the basis of color vision in zebrafish has been addressed in a number of studies (Meier et al., 2018; Zimmermann et al., 2018; Baden et al., 2019; Baden, 2021), it remains difficult to find a fully conclusive explanation, especially as the exact spectrum of the projected color cannot be controlled with our current setup. In the context of regeneration, we applied a two level vision test. The first level probes for visual performance under different difficulty levels defined by varying combinations of contrast and spatial frequency. Consistent with our social preference test, we find a major visual impairment immediately after light lesion (Figure 5). Subsequently, we observe that the OKR is restored in the course of regeneration. Importantly, restoration occurs gradually with easier stimuli being already tracked sufficiently after 10 days whereas more demanding stimuli are only resolved at 14 days after light lesion. A direct comparison of the relative tracking efficiency over time for the three degrees of difficulty confirmed this finding. The second level of the vision test probes for the perception of colors. Consistent with our social preference test and the first level vision test we observe that the perception of colors is impaired immediately following a light lesion (Figure 6). Subsequently, color vision is fully restored within 10 days indicating that the ability to perceive colors is less elaborate compared to other visual functions because it occurs in the same time frame as the resolution of easier stimuli in the first level vision test. We conclude that contrast sensitivity, spatial resolution and also perception of colors are fully regained after lesion and that the kinetics of recovery is dependent on the degree of difficulty of the visual stimulus.

In contrast to the fast functional recovery of our applied light lesion paradigm, the recovery of vision-dependent behavior after neurotoxic lesion requires up to 100 days depending on the degree of damage (Sherpa et al., 2008; Sherpa et al., 2014). However, reintegration of newly regenerated photoreceptors into an existing circuity might be less complex and therefore faster in comparison to a full re-establishment of a retinal network following a severe damage caused by neurotoxins. Consistent with that, morphological recovery after neurotoxic lesion takes up to 60 days whereas morphological regeneration is already completed at 28 days post light lesion (Weber et al., 2013; Sherpa et al., 2014). In accordance, it has been shown that the OKR is restored within 15 days after Methylnitrosurea-induced ablation of rod photoreceptors (Maurer et al., 2014).

As indicated above, regeneration is a complex interplay of structural and functional recovery. Hence, we investigated the underlying morphological processes at the critical time points of vision recovery. Optical coherence tomography (OCT) imaging has proven useful to monitor retinal regeneration before (Bailey et al., 2012; Bell et al., 2016; Huckenpahler et al., 2020) as it allows a detailed analysis of retinal structures *in vivo* (Figure 7). We found a close correlation between the kinetics of morphological regeneration and functional restoration of vision. At 7 dpl, when recognition of motion and form of conspecifics are already restored, we observe first cells in the outer nuclear layer. In addition, inner and outer segments of photoreceptors are reappearing indicated by the hyper-reflective dots in the OCT images. At 10 and 14 dpl when also color perception, contrast sensitivity and spatial resolution are regained, the outer nuclear layer became more organized and the outer segments of the photoreceptors had grown in size. Finally, at 28 dpl when all parameters of the OKR analysis have reached pre-lesion levels, the retinal structure was largely restored, although the photoreceptor mosaic appeared less organized compared to the unlesioned retina. This phenomenon has already been described together with other regeneration-specific phenotypes including patterning abnormalities in retinal lamination as well as the generation of supernumerary neurons (Fraser et al., 2013; Sherpa et al., 2014; Powell et al., 2016; Ranski et al., 2018). On a molecular level, a recent study described a transcriptional shift from progenitor proliferation to cell differentiation between 5 and 10 days after light lesion (Kramer et al., 2021) which is in line with our observations on structural and functional recovery. They also show that the expression levels of all four opsins and rhodopsin, the photosensitive molecules of the retina, were restored to pre-lesion levels at 10 dpl. Along this line, they report the expression of blue opsins in the newly regenerated photoreceptors supporting our finding that the perception of colors is restored at the same time point. Together, we conclude that performance in less demanding vision-controlled tests is already restored before the final maturation of photoreceptor cells, indicating a fast integration of regenerated cells into the existing circuity and their early functionality. More detailed histological studies looking into the morphology of photoreceptors with a special focus onto their inner and outer segments will be essential to understand the structural requirements for photoreceptors to mediate visual function.

The presented combination of *in vivo* imaging and behavioral assays will allow to analyze structural as well as functional recovery in the very same animal for various scientific questions. In this respect, it will be of great interest to compare the kinetics of vision recovery in different lesion models. In neurotoxin-mediated lesions, the degree of morphological recovery can vary greatly between individual fish and the correlation of morphological and functional data will be key to provide further insights into the morphological prerequisites required for different visual functions (Sherpa et al., 2008; Sherpa et al., 2014). With recent advances in zebrafish transgenesis and genome engineering, more sophisticated models for human retinal degenerative diseases will be available (Richardson et al., 2017; Zheng et al., 2018). Following the progressive loss of vision in individual fish and correlating accompanying changes in structure and function will enable new insights into disease progression as well as into their potential therapeutic interventions. Finally, the combined analysis of structure and function will be interesting to study visual performance in the context of ageing as recently an age-related neurodegenerative phenotype has been described (Van houcke et al., 2019).

Vision is the ultimate output of retinal activity and hence the restoration of visual function is most relevant with regard to the treatment of human retinal degenerative diseases. Here, we show that vision-controlled behavior is restored in adult zebrafish within a short time frame after light lesion and that contrast sensitivity, spatial resolution and the perception of colors are regained. With regard to the morphological data, we reveal a considerable correlation between the regeneration of structure and function and suggest that restoration of visual performance can be observed already before full morphological restoration of the tissue. However, further studies are required to address the functionality of individual regenerated cells as well as their functional integration into the existing circuity. The electrical activity of whole retina explants upon light stimulation as an objective measure of retinal function on the tissue level can be recorded using electroretinography in adult zebrafish (Nadolski et al., 2021). Mensinger and colleagues have shown that the electrical activity of the retina in goldfish is gradually recovered but not fully restored within 200 days after surgical lesion or within 70 days after Ouabain injection (Mensinger and Powers, 1999, Mensinger and Powers, 2007). In contrast, a more recent study described the functional re-establishment of the photoreceptor-bipolar cell connectivity accompanied by the full restoration of the electroretinogram (ERG) within 60 days in the context of a neurotoxin-mediated lesion (McGinn et al., 2018). A detailed morphometric analysis of regenerated bipolar cells and their dendritic trees revealed that the characteristics of regenerated bipolar interneurons resemble the ones of mature neurons, but also a high degree of morphologically abnormal bipolar cells after regeneration (McGinn et al., 2019). It remains to be studied if these abnormal cells contribute to retinal function and if functional rewiring is required for restoration of high acuity vision. Indeed, calcium imaging would allow the detailed functional analysis of individual cells and has been successfully applied in larval zebrafish (Zimmermann et al., 2018) but remains challenging in the adult retina (Angueyra and Kindt, 2018). Altogether, a clearer understanding of the regenerative processes will contribute significantly to further advance treatments of human retinal degenerative diseases.

## Data Availability

The original contributions presented in the study are included in the article/Supplementary Material, further inquiries can be directed to the corresponding author.

## Abbreviations

cpd, cycles per degree; dpl, days post lesion; dps, degrees per second; HE, hematoxylin/eosin; OCT, optical coherence tomography; OKR, optokinetic response; ROI, region of interest.
