# Supplementary figures and images for: Visual Function is Gradually Restored During Retina Regeneration in Adult Zebrafish
Source: Front Cell Dev Biol. 2022 Feb 1;9:831322. doi: 10.3389/fcell.2021.831322 (PMC8844564; doi:10.3389/fcell.2021.831322)

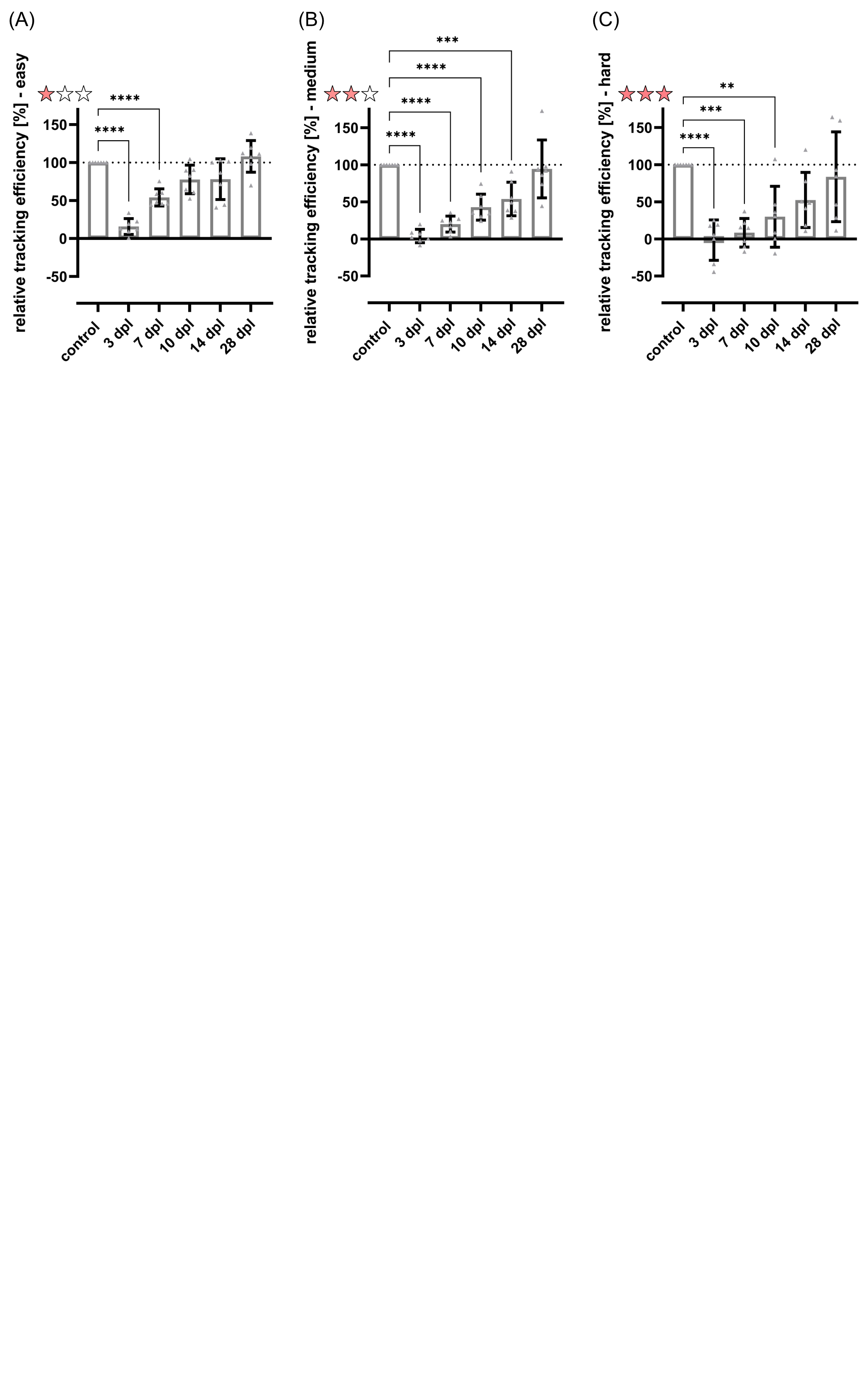

Supplement: Supplementary file 1 [file Image3.TIFF]

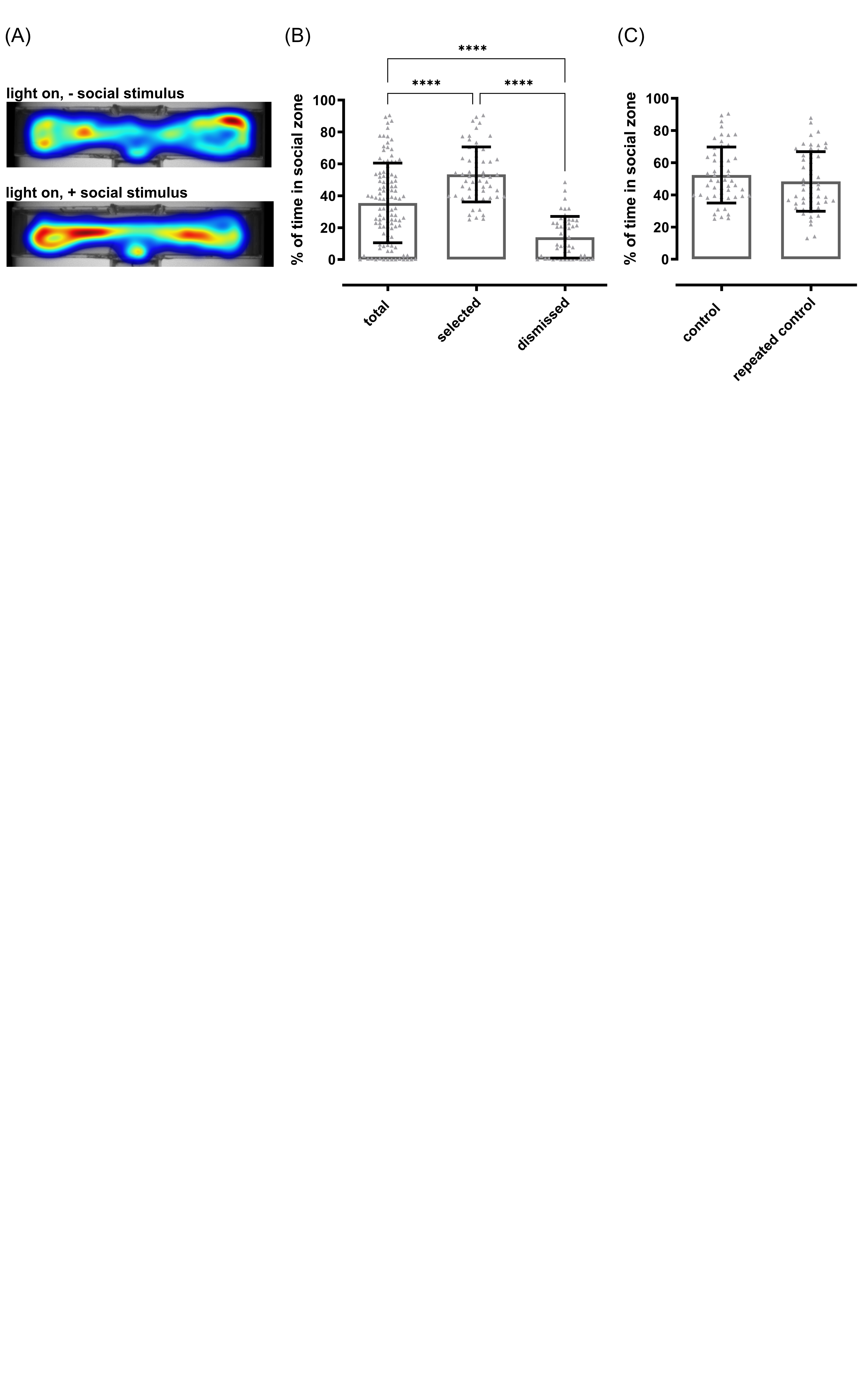

Supplement: Supplementary file 2 [file Image1.TIFF]

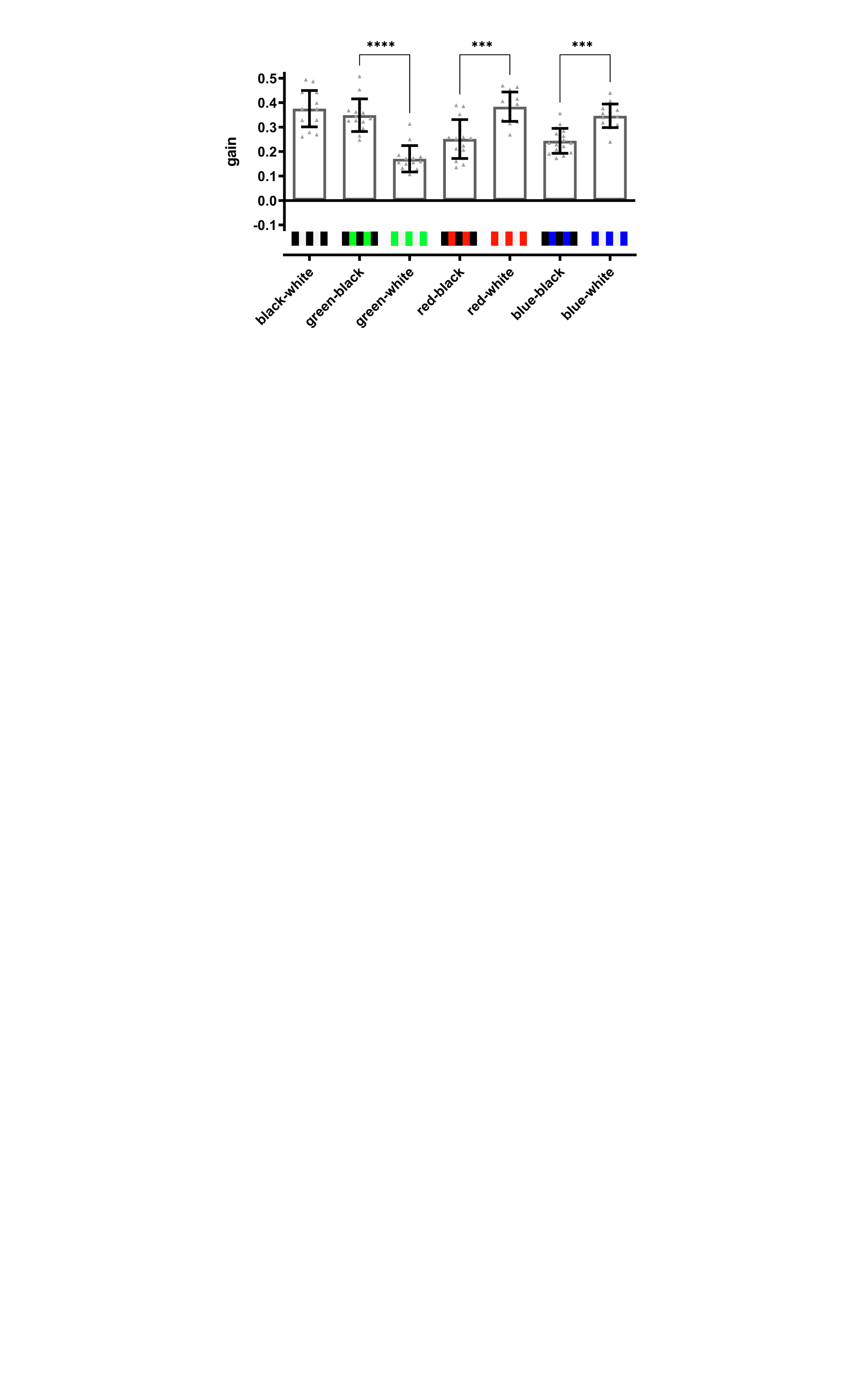

Supplement: Supplementary file 3 [file Image2.TIFF]

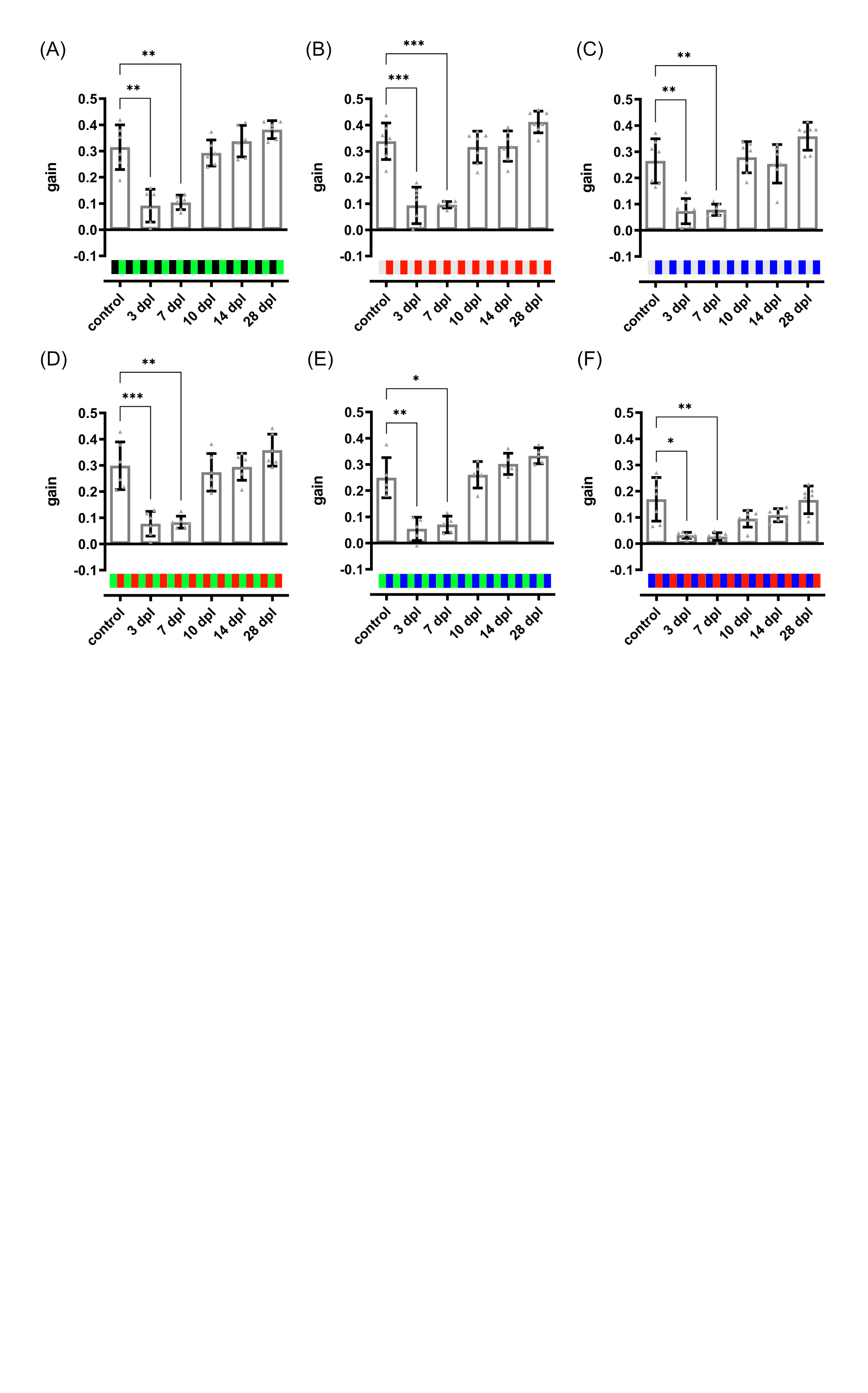

Supplement: Supplementary file 4 [file Image4.tiff]
