# Supplementary material for: Visual Function is Gradually Restored During Retina Regeneration in Adult Zebrafish
Source: Front Cell Dev Biol. 2022 Feb 1;9:831322. doi: 10.3389/fcell.2021.831322 (PMC8844564; doi:10.3389/fcell.2021.831322)
Supplement: Supplementary file 5 [file DataSheet1.docx]

**Figure S1: The social preference is variable between fish but remains stable for individual fish.** **(A)** Heat maps of an exemplary “unsocial” animal showing the average location of the fish in the presence (light on, + social stimulus) and absence (light on, - social stimulus) of conspecifics in the second compartment. Even in the presence of a social stimulus, the animal kept roaming the whole compartment and did not spend much time in the social zone. **(B)** Quantification of the time spent in the social zone revealed a huge variation in the preference of individual fish for a social stimulus (35.5 ± 24.9 %). Fish were subsequently sorted for the study of functional recovery based on the time spent in the social zone as well as general swimming parameters. This way, animals were either selected (53.4 ± 17.1 % of fish) or dismissed (14 ± 12.9 % of fish) for further experiments. **(C)** Comparison of the quantifications of the time the selected fish spent in the social zone in the initial recording (control) and 7 days later (repeated control) showed no significant change (52.3 ± 17.3 % versus 48.4 ± 18.4 %). Statistics: all data are represented as mean ± SD, one-way ANOVA with Tukey’s multiple comparison test, p < 0.05 (*); 0.01 (**); 0.001 (***) or 0.0001 (****).

**Figure S2: The tracking of colors in the optokinetic response test is dependent on the selected background color.** Quantification of the gain obtained with colored stripes on white versus black background revealed significant changes. Green stripes with black background (0.35 ± 0.06) resulted in a twice as high gain as compared to white background (0.17 ± 0.05). Red or blue stripes combined with a white background (red: 0.38 ± 0.06, blue: 0.35 ± 0.05) elicited an increased gain compared to a black background (red: 0.25 ± 0.08, blue: 0.24 ± 0.05). The gains observed for black-white (0.38 ± 0.07), green-black, red-white and blue-white stripes were comparably high. Statistics: all data are represented as mean ± SD, paired t-test color-black vs. color-white, p < 0.05 (*); 0.01 (**); 0.001 (***) or 0.0001 (****).

**Figure S3: The OKR-based first level vision test reveals differences in the kinetics of functional recovery dependent on the degree of difficulty.** **(A-C)** Quantifications of the relative tracking efficiency (gain normalized to pre-lesion levels for each individual fish and subsequently pooled for all conditions of a certain degree of difficulty) showed a highly significant reduction at 3 and 7 dpl for all three conditions (easy, medium and hard indicated with one, two and three red stars, respectively) (see supplementary table 1). For easy conditions, no statistical difference was observed from 10 dpl onwards indicating recovery of vision. In contrast, loss of a statistical difference was seen for the medium and hard conditions only at 28 and 14 dpl, respectively. Statistics: all data are represented as mean ± SD, one-way ANOVA with Dunnett’s multiple comparison test against control, p < 0.05 (*); 0.01 (**); 0.001 (***) or 0.0001 (****).

**Figure S4: The perception of colors on black or white background as well as two colored stimuli is restored within 10 days after light lesion.** Quantifications of the gain obtained with green-black **(A)**, red-white **(B)**, blue-white **(C)**, red-green **(D)**, green-blue **(E)** and blue-red **(F)** stimuli with constant contrast (100 %), spatial frequency (0.2 cpd) and angular velocity (15 dps) prior to lesion (control) and at 3, 7, 10, 14 and 28 days post lesion (dpl), showed a highly similar result. With all stimuli, a significantly reduced gain were observed at 3 and 7 dpl which was restored to pre-lesion levels at 10 dpl (see supplementary table 2). Statistics: all data are represented as mean ± SD, one-way ANOVA with Dunnett’s multiple comparison test against control, p < 0.05 (*); 0.01 (**); 0.001 (***) or 0.0001 (****).

**Table S1: Values of the relative tracking efficiency during the course of regeneration.**

| degree of difficulty | relative tracking efficiency [%] | | | | |
| --- | --- | --- | --- | --- | --- |
|  | 3 dpl | 7 dpl | 10 dpl | 14 dpl | 28 dpl |
| easy | 15.9 ± 9.7 | 54.1 ± 10.5 | 77.8 ± 17.5 | 78.3 ± 24.8 | 108.2 ± 19.4 |
| medium | 3.9 ± 8.4 | 20 ± 9.9 | 42.9 ± 16.2 | 54 ± 21 | 94.4 ± 36.2 |
| hard | -1.5 ± 25.2 | 8.4 ± 17.8 | 29.9 ± 38 | 52.4 ± 34.4 | 83.8 ± 56 |

Abbreviations: dpl – days post lesion.

**Table S2: Individual data points for color vision in the OKR-based vision test during the course of regeneration.**

| OKR parameters | | | | gain | | | | | |
| --- | --- | --- | --- | --- | --- | --- | --- | --- | --- |
| color | contrast  [%] | SF  [cpd] | AV  [dps] | control | 3 dpl | 7 dpl | 10 dpl | 14 dpl | 28 dpl |
| green - black | 100 | 0.2 | 15 | 0.31 ± 0.08 | 0.09 ± 0.06 | 0.1 ± 0.02 | 0.29 ± 0.05 | 0.34 ± 0.05 | 0.38 ± 0.03 |
| red - white | 100 | 0.2 | 15 | 0.34 ± 0.06 | 0.09 ± 0.06 | 0.1 ± 0.01 | 0.32 ± 0.06 | 0.32 ± 0.05 | 0.41 ± 0.04 |
| blue - white | 100 | 0.2 | 15 | 0.27 ± 0.08 | 0.07 ± 0.04 | 0.08 ± 0.02 | 0.28 ± 0.05 | 0.25 ± 0.07 | 0.36 ± 0.05 |
| red - green | 100 | 0.2 | 15 | 0.3 ± 0.08 | 0.08 ± 0.04 | 0.08 ± 0.02 | 0.27 ± 0.07 | 0.29 ± 0.05 | 0.36 ± 0.06 |
| blue - green | 100 | 0.2 | 15 | 0.25 ± 0.07 | 0.05 ± 0.04 | 0.07 ± 0.03 | 0.26 ± 0.05 | 0.3 ± 0.04 | 0.33 ± 0.03 |
| red - blue | 100 | 0.2 | 15 | 0.17 ± 0.08 | 0.03 ± 0.01 | 0.03 ± 0.01 | 0.1 ± 0.03 | 0.11 ± 0.02 | 0.17 ± 0.05 |

Abbreviations: AV – angular velocity; cpd – cycles per degree; dpl – days post lesion; dps – degrees per second; SF – spatial frequency.
